# Do dominant group members have different emotional responses to observing dominant-on-dominant versus dominant-on-disadvantaged ostracism? Some evidence for heightened reactivity to potentially discriminatory ingroup behavior

**DOI:** 10.1371/journal.pone.0234540

**Published:** 2020-06-25

**Authors:** Corey Petsnik, Jacquie D. Vorauer

**Affiliations:** Department of Psychology, University of Manitoba, Winnipeg, Manitoba, Canada; Ghent University, BELGIUM

## Abstract

The importance of social connection to well-being is underscored by individuals’ reactivity to events highlighting the potential for rejection and exclusion, which extends even to observing the social exclusion of others (“vicarious ostracism”). Because responses to vicarious ostracism depend at least in part on empathy with the target, and individuals tend to empathize less readily with outgroup than ingroup members, the question arises as to whether there is a boundary condition on vicarious ostracism effects whereby individuals are relatively immune to observing ingroup-on-outgroup ostracism. Of particular interest is the case where members of a dominant ethnic group observe fellow ingroup members ostracize a member of a disadvantaged ethnic minority group, as here there is a compelling potential alternative: Perceived violation of contemporary social norms condemning prejudice and discrimination might instead lead dominant group members to be especially upset by “dominant-on-disadvantaged” ostracism. Accordingly, the present research examines, across four studies and 4413 participants, individuals’ affective reactions to observing dominant-on-disadvantaged versus dominant-on-dominant ostracism. In each study, dominant group members (White/Europeans) observed dominant group members include or ostracize a fellow dominant group member or a disadvantaged ethnic minority group member (a Black individual) in an online Cyberball game. Results revealed that dominant group members felt more guilt, anger, and sadness after observing severe ostracism of a disadvantaged as opposed to dominant group member. Although no direct effects emerged on behavioral outcomes, exploratory analyses suggested that observing ostracism of a disadvantaged (versus dominant) group member had indirect effects on behavior via increased feelings of anger. These results suggest that observing ostracism may be a sufficiently potent and relatable experience that when it occurs across group boundaries it awakens individuals’ sensitivity to injustice and discrimination.

## Introduction

Social connections are profoundly important to mental and physical well-being [1]. Moreover, relations and interactions with others help ground a fundamental aspect of individuals’ self-perception: Individuals’ own feelings of self-esteem are heavily intertwined with their feelings of being socially accepted or rejected [2]. Consequently, individuals are highly sensitive to information that even only remotely reflects on their social standing with others. Attesting to the deeply ingrained and automatic nature of such sensitivity, it applies even in cases where negative treatment is plausibly directed toward others instead of oneself, or comes from an unimportant or despised other or a computer [3–7].

This sensitivity goes further yet: Individuals who observe another person being ostracized–even when there is no potential for personally being a target of mistreatment themselves–experience reduced satisfaction of fundamental needs (e.g., belonging and self-esteem) and more negative mood [8]. Such “vicarious ostracism” has been found across observers of various ages, ranging from children to adults [9] and at times appears to be just as potent as being directly targeted [10].

Beyond these intrapersonal effects, vicarious ostracism has also been linked to a variety of interpersonal outcomes. In particular, observing ostracism may often motivate prosocial behavior by observers toward the targeted individual, perhaps with the intention of alleviating their distress. For instance, observers actively seek to reinclude ostracized individuals, write excluded others encouraging emails, and in economic games sacrifice some of their own resources to compensate the target [8,9,11,12]. Moreover, in addition to assisting ostracized individuals, studies have found that observers will also punish the perpetrators (e.g., by allocating them less money [8,9,13,14]).

However, it is not always the case that individuals are distressed by observing ostracism and subsequently act to assist the target and discipline the perpetrators. Indeed, research indicates that under certain conditions observers are not only unperturbed by a target’s ostracism, reporting, for instance, feeling little anger about the target’s mistreatment or sympathy toward him or her, but are also motivated to blame and punish the target, and may even join the perpetrators in ostracizing them [15–19].

The present research aims to further elucidate some of the factors that might make observers more or less sensitive to witnessing the ostracism of another individual. Specifically, we examine the effect of a target’s ethnic group membership on observers’ reactions to ostracism, with a particular focus on how dominant ethnic group members react to observing other dominant group members perpetrate ostracism against a disadvantaged ethnic minority group member (“dominant-on-disadvantaged” ostracism) relative to a fellow dominant group member (“dominant-on-dominant” ostracism). Combining theory and research on the effect of group membership on observers’ reactions to ostracism with that on intergroup perception, affect, and relations we propose three alternative hypotheses for how observers might react to dominant-on-disadvantaged versus dominant-on-dominant ostracism: Reduced reactivity, enhanced reactivity, or equal reactivity. We then test these competing hypotheses in a “mini-meta-analysis” [20] across four online experiments.

### Reduced reactivity hypothesis

Research indicates that vicarious ostracism is enhanced when individuals actively try to take the perspective of the target [21] or are high in dispositional empathy [22]. These findings point to a readiness to identify and empathize with targets of ostracism as a key underlying mechanism. The important role played by empathy in driving individuals’ reactivity to others’ mistreatment also suggests a potential boundary condition: Perhaps when the witnessed ostracism involves ingroup members ostracizing an outgroup member individuals’ sensitivity is shut down. Such reduced reactivity would make sense from a variety of perspectives. Most notably, ample research suggests an “empathy gap” across group boundaries whereby individuals are less likely to empathize with outgroup than ingroup members in a range of different circumstances, such as when observing another person’s expressions of sadness [23], witnessing another individual’s experience of physical pain [24,25], or learning of negative events or misfortunes another person has experienced [26]. Deficits in empathy have been identified across various naturally occurring intergroup boundaries (e.g., political or ethnic differences) as well as boundaries created in the laboratory, indicating that an empathy gap for outgroup members is a reliable and robust phenomenon [23,27–29]. Together with research indicating an important role for empathy in vicarious ostracism, this evidence for an empathy deficit with respect to outgroup members, including those that may belong to a different and potentially disadvantaged ethnic group [30], suggests that dominant group members should have less negative reactions to seeing dominant group members ostracize a disadvantaged group member as compared to a fellow dominant group member.

Other forces may also contribute to dominant group members' reduced reactivity to observing dominant-on-disadvantaged ostracism. In particular, the motivation to maintain a positive social identity should presumably push these individuals toward downplaying the negativity of the target’s treatment, perhaps by considering it as provoked or justified [31]. Relatedly, dampened reactions may be particularly evident when the observer and perpetrators share an advantaged group membership, the target is a member of a disadvantaged group, and the former perceive their advantage as legitimate [32,33]. This may be especially likely if advantaged observers feel that as result of their association with the perpetrators they are being unfairly accused of being prejudiced [34].

The hypothesis that dominant group members will react less negatively when witnessing ingroup members ostracize a disadvantaged group member as compared to a fellow dominant group member, which we refer to as the *reduced reactivity hypothesis*, is also in line with research suggesting bystander apathy, in both conscious self-reports and physiological responding, with respect to witnessing explicitly racist and homophobic comments directed toward outgroup targets [35–37].

Although only limited research has directly evaluated the impact of group membership on observers’ reactions to ostracism, the results of some of these studies provide support for the hypothesis that dominant group members will be less distressed by witnessing dominant-on-disadvantaged relative to dominant-on-dominant ostracism. Notably, Veldhuis et al. [38] demonstrated that witnessing the ostracism of those who share one’s political preferences elicits greater feelings of humiliation relative to observing the ostracism of those with differing political leanings. Similarly, manipulating ingroup-outgroup status using a minimal group paradigm, Forbes et al. [39] found that observers were more likely to reinclude an ostracized ingroup member relative to an outgroup member. Additional research documenting that a peer relationship or close friendship with targets enhances observers’ empathic responses toward ostracized targets [40,41] and that a peer relationship or close friendship with perpetrators diminishes observers’ punishment of perpetrators [13,42], as well as work indicating that targets experience greater distress when they are ostracized by racial ingroup relative to outgroup members [43,44], is also broadly supportive of reduced reactivity to dominant-on-disadvantaged ostracism.

### Enhanced reactivity hypothesis

However, a contrasting possibility, enhanced reactivity (*enhanced reactivity hypothesis*), is also supported by several lines of research and theorizing. Perceived violation of contemporary social norms condemning prejudice and discrimination [45] may lead individuals to be more upset by ostracism across as compared to within group boundaries. Enhanced perceptions of the unfairness and pervasiveness [46] of group-based treatment could conceivably contribute to such effects, perhaps especially in the case of ascribed characteristics such as sex and race. Negative reactions could be even more likely on the part of dominant group members when the ostracism is perpetrated by ingroup members against a member of an ethnic minority group because of the social identity threat represented by the possibility that their ingroup–and by extension they themselves–are racist or appear to be racist [47–49]. Consistent with this idea, witnessing but not confronting discriminatory behavior has been shown to be a potentially dissonance-arousing experience [50]. Further, prior research suggests that the salience of a dominant group identity may elicit a variety of negative group-based emotions if individuals appraise their ingroup as responsible for perpetrating harm or illegitimate advantage over an outgroup [51,52].

Some research on the impact of group membership on observers’ responses to ostracism also suggests heightened reactivity in the intergroup case. In particular, Rudert et al. [16] proposed the “social dissimilarity rule” whereby observers’ reactions to witnessing ostracism are governed by perceived similarity or dissimilarity between targets and perpetrators, such that when individuals observe exchanges in which a dissimilar person is ostracized they are inclined to devalue the ostracizers, sympathize with the target, and attribute the ostracism to malicious motives (e.g., prejudice or discrimination), whereas if a similar person is ostracized they devalue the target and attribute the ostracism to punitive motives. Rudert et al. found support for this hypothesis across a series of studies where similarity/dissimilarity was manipulated through shared group membership, including shared ethnic background. However, because in these studies participants did not share group memberships with ostracizers or targets–which could clearly affect their identifications and motivations–the implications for situations, such as dominant-on-disadvantaged ostracism, where ingroup members are observed ostracizing an outgroup member are unclear.

In another study examining observers’ judgments of ostracism more generally, Rudert et al. [53] found that the ostracism of individuals perceived as warm-and-incompetent was judged to be the least acceptable. As members of many disadvantaged groups tend to be viewed as more warm than competent [54], observers’ general beliefs about the attributes of a disadvantaged target may lead them to especially condemn their mistreatment. Further, research on targets’ reactions has revealed more reactivity to outgroup versus ingroup ostracism, at least on some measures. For instance, Schaafsma et al. [55] found that exclusion by outgroup members led to more hostility than exclusion by ingroup members, an effect that was mediated by attributions to racism, and Williams et al. [56] found that being ostracized by outgroup members reduced felt belonging more than did being ostracized by ingroup members.

### Equal reactivity hypothesis

Of course another possibility is that the target’s group status just does not matter to individuals’ reactions. This could suggest that ostracism is such a fundamental and universal experience that observing it take place is sufficiently powerful and basic to overwhelm any effects associated with whether the target is an ingroup or an outgroup member. Perhaps when individuals can readily feel that “it could happen to me” they are poised to identify with and react to another’s experience on its own terms irrespective of the person’s group membership. Broadly consistent with this possibility is research by Arpin et al. [57] that found, using a minimal group paradigm, no impact of group membership on observers’ reactions to ostracism. Also relevant here is work on targets’ responses indicating similar levels of distress regardless of whether the perpetrators are ingroup or outgroup members [4,58,59].

## Overview

We probed dominant group members’ reactions to dominant-on-dominant vs. dominant-on-disadvantaged ostracism in a series of four online experiments involving a total of 4413 participants. In each of the experiments White participants observed a computer-mediated ball-tossing game (“Cyberball” [56]) in which two White individuals either ostracized or included a third player, who was either a White or Black individual. Their affective and behavioral reactions and impressions of the players were then assessed.

Several of our design decisions warrant discussion. First, because our theoretical focus was on the implications of the target’s group membership for observers’ reactions to ostracism, it was necessary to hold the perpetrators’ group membership constant. Thus we considered dominant-on-dominant ostracism to be the most appropriate control condition against which dominant-on-disadvantaged ostracism could be compared. This comparison afforded by our studies is relatively unique. To our knowledge only one other study has compared ingroup-on-ingroup to ingroup-on-outgroup ostracism, that of Forbes et al. [39], and they did so using a minimal group paradigm. Indeed most previous work–which was focused on answering different questions than those of interest here–has either not considered the effects of a shared group membership between observers, ostracizers, and targets [16] or has probed the effects of observing outgroup-on-ingroup versus outgroup-on-outgroup ostracism [38,57]. Although this research yielded valuable insights, it does not speak clearly to the effect of the target’s group membership on reactions to ingroup-perpetrated ostracism, which is of central interest in the present studies.

Second, we focus on the effects of ethnic group membership and the case of dominant group members’ ostracism of a disadvantaged ethnic minority group member target in particular–as opposed, for example, to group memberships based on political [38] or computer preferences [56] or those induced experimentally through minimal group paradigms [39,57]. Our focus on this specific case sets our studies apart from past work. Notably, basing group membership on distinctions, such as those instantiated by minimal group paradigms, that are relatively minor, as opposed to distinctions, like ethnic background that are especially salient, essentialized, and affectively charged may account for some of the conflicting or null effects of group membership on observers’ reactions to ostracism. From a social justice perspective, understanding how members of powerful groups react–or fail to react–to the negative treatment of members of historically disadvantaged groups has the potential to illuminate mechanisms through which group power relations are reinforced and perpetuated, or more optimistically, conditions under which dominant group members are more sensitive to injustice.

Our primary dependent measures were participants’ affective reactions to the behavior they observed: Their feelings of guilt, anger, sadness, and fear as well as empathy for the target were assessed, along with general positive affect (as a filler). However, we also considered the implications for individuals’ evaluations of the ostracized target. Of particular interest was whether, as a result of dissonance-related processes, any enhanced reactivity to dominant-on-disadvantaged ostracism might coincide with more negative evaluations of disadvantaged group targets. Specifically, to the extent that individuals experience a social identity threat as a result of witnessing the potentially racist behavior of fellow dominant group members they may react defensively by devaluing or disparaging the disadvantaged target. Indeed, a large body of research indicates that various self as well as social identity threats may motivate defensive reactions ranging from the subtle denial of uniquely human emotions to outgroup members (i.e., infrahumanization [60]) to outright derogation [61–64]. Accordingly, several items assessed participants’ evaluations of the target. Finally, compensatory behavior toward targets and punishment behavior toward perpetrators were assessed as potential downstream consequences of negative affect.

Although most past work examining vicarious ostracism has typically evaluated the extent to which observers themselves feel excluded by assessing, for instance, their need satisfaction (e.g., feelings of belonging and self-esteem), we forwent this approach in the present studies. We were less focused on the intrapersonal consequences of witnessing ostracism and more on its interpersonal implications. For that reason, the affective reactions we measured were those, like anger, guilt, and empathy that might be most likely to motivate observers’ behavioral responses toward the target and perpetrators. Further, observers’ feelings of empathy toward the target might heavily overlap with and operate similarly to their feelings of vicarious exclusion. Our focus on motivating affective responses is aligned with prior research that has sought to evaluate the impact of group membership on observers’ responses to witnessing ostracism rather than assessing reactions irrespective of group membership [16,39].

Although we have outlined theoretical bases for different potential patterns of results for affective reactivity, we refrain from expressing specific predictions here regarding which pattern will be most evident or regarding specific mediational chains. Our intellectual journey to the present theoretical framing of this research has been circuitous, as documented in two pre-registrations that can be accessed at osf.io/9jk3c and osf.io/ng796. Perhaps most notably, we initially had distinct predictions for observing moderate versus severe ostracism and our initial predictions did not focus on specific negative affective states whereas our later ones did. Here we combine together all of the studies that we have conducted to date on this topic, which follow a very similar methodology, and conduct a “mini-meta-analysis” [20] to evaluate the weight of the evidence overall for the reduced or enhanced (or equal) reactivity hypotheses using two-tailed significance tests. In line with our most recent pre-registration (osf.io/ng796), we also test whether effects obtained on behavioral reactions are mediated by affective reactions in an exploratory manner, with the type of reaction tested determined in a data-driven manner by the pattern of results obtained across the various measures.

We now proceed to outline in full the design and procedures of each of the four studies, except that for the dependent measures we only report our focal measures, namely participants’ affective reactions, their feelings of empathy toward the target, as well as their overall impressions of him or her. In addition, we also report results for Study 4 because this study assessed several behavioral responses that were not assessed in the other studies and thus provides a unique opportunity to test indirect effects on behavior. All additional measures not included in the main text are listed in the (S1 File), as are results for Studies 1 to 3. After outlining the method for each of the studies as well as the results for Study 4, we then report the meta-analysis across all four studies and discuss the combined results.

## General method

In each study participants were either introductory psychology students or Amazon Mechanical Turk workers, all with a White/European ethnic background who completed an online study of social perception in exchange for partial course credit or 0.50 USD. Studies 1 and 2 sampled only women, whereas Studies 3 and 4 also sampled men. For each individual study sample size was determined in a priori power analyses (α = .05, two-tailed) before any data analysis. Given initial uncertainty about the size of expected effects when we began this program of research, the sample size for Study 1 was selected to provide .95 power to detect the average effect size in social psychology found by Richard et al. ([65]; *f* = 0.21). In contrast, for Studies 2 through 4, sample size was selected to provide .80 (Study 2) to .85 power (Studies 3 and 4; see pre-registrations for details) to detect a small interaction effect size (*f* = 0.10–0.11). However, for consistency across studies we report the outcome of sensitivity power analyses for each study indicating the effect size we had .80 power to detect rather than the results of a priori power analyses because the a priori power analyses used to determine the sample sizes for Studies 3 and 4 included accommodations for attrition due to exclusions not implemented in the analyses reported in this paper. A summary of sample characteristics for all studies is provided in Table 1.

**Table 1:** Sample characteristics.

Study	Sample	*N*	% female	Age (years) *M SD*
1	Psych students	301	All	19.90 4.73
2	MTurk	924	All	39.01 13.25
3	MTurk	1958	65.2%	36.55 11.60
4	MTurk	1230	65.7%	36.86 11.93
Total	4413			74.9% women	33.08 10.38

Psych students = introductory psychology student pool; MTurk = Amazon Mechanical Turk; All participants reported a White/European ethnic background.

In each study participants were randomly assigned to observe the inclusion or ostracism of another ostensible participant in a game of ball-toss by two other ostensible participants, after which their affect, empathy, and impressions were assessed. The game of ball-toss consisted of Cyberball [66], a paradigm widely used to evaluate the effects of directly experiencing ostracism, but which can, by implementing the observer role, be used to examine the effects of witnessing ostracism. In the version of Cyberball we employed, the three players were represented by cartoon avatars and photographs and appeared to toss a ball amongst themselves while the participant watched. Although participants were informed that they and the other participants were randomly assigned to be either players or observers, in reality there were no other participants, all participants were assigned the role of observers, and the behavior of the players was entirely computer-programmed. All studies included a *severe ostracism* and an *inclusion* condition. In the severe ostracism condition, participants witnessed the target receive only two out of 30 total tosses (6.67% of the throws) from the other players near the beginning of the game and then never again for the remainder of it. In the *inclusion* condition, the target, along with the other players, received an equal 10 total tosses (33.33% of the throws). In addition, Studies 2 and 3 also included a *moderate ostracism* condition in which the target received six out of 30 tosses (20% of the throws) at the start of the game. In all studies, across observed treatment conditions, target type was operationalized by ethnic background and was manipulated through the photos apparently uploaded by the participants ostensibly selected as players. In *White/European target* games the photo uploaded by the target portrayed an individual with a White/European ethnic background. In *Black target* games the photo uploaded by the target portrayed an individual with a Black ethnic background. In all games the photos ostensibly uploaded by the perpetrators were of individuals with White/European ethnic backgrounds. These photos were obtained with permission from contacts of the first author and were taken in a candid and realistic “selfie” style to enhance the credibility of the cover story. The photos presented were matched to participants’ gender, but their order and role were not counter-balanced.

As per our pre-registrations, in each study participants were excluded if they: a) did not complete any of the main dependent variables; b) completed the survey, in part or in full, more than once (only duplicate responses beyond the first submitted were excluded); c) indicated a non-White/European ethnic background or a sex other than male or female, or d), were not able to view the manipulation.

However, we deviate from our first pre-registration in two ways. First, our first pre-registration indicated that we would also exclude participants who failed to correctly answer attention-check items or incorrectly answered manipulation check items regarding the ethnicity of the players and the type of observed treatment (indicating whether one person received next to no ball tosses or all players received the ball about equally, or, in Studies 2 and 3, one person received somewhat fewer tosses than the others). Because the exclusion criteria were arguably overly stringent (especially regarding distinguishing between moderate versus severe ostracism) and vulnerable to social desirability motivations (most notably a desire to appear “color-blind” and not notice player ethnicity), we abandoned these going forward to minimize participant attrition and avoid introducing bias into the sample [67].

Likewise, although our first pre-registration indicated a plan to exclude participants who indicated familiarity with or suspicion about the authenticity of Cyberball, we did not exclude on suspicion in any of the analyses reported in the main text. When we evaluated the robustness of the meta-analytic effects to participant suspicion, by coding whether participants indicated suspicion in an open-ended thought-listing task that immediately followed the game of Cyberball in Studies 2 to 4 and then conducting the meta-analysis with these participants excluded, we found that the focal pattern of results obtained across the studies was essentially unaffected. In view of these results, and because we sought to minimize participant attrition, we opted not to exclude potentially suspicious participants in the final analyses reported in the main text. We further note that research revealing that direct experiences with ostracism are still aversive even when people know their treatment was computer generated or scripted [5] suggests that doubts about the authenticity of the manipulation do not fully undermine its effectiveness. Additional details regarding the coding, analyses, and results regarding participant suspicion are provided in the (S1 File).

### Ethics statement

All studies were approved by the Research Ethics Board at the University of Manitoba: Approval number #P2016:150 (HS20351). All participants provided consent by clicking on a button on an (online) written consent form.

## Study 1

### Method

#### Participants

A sensitivity power analysis indicated that the sample size of 301 would provide .80 power to detect an effect of *f* = 0.162 ($\eta_{p}^{2}$ = .013).

#### Procedure and design

Participants connected to the study website for a study ostensibly examining social perception processes and were instructed that they would engage in an online game of ball-toss (i.e., Cyberball) with three other people who were also currently participating in the study. After being notified that they had been selected for the role of observer, participants were randomly assigned to one cell of a 2 (observed treatment: severe ostracism vs. inclusion) × 2 (target type: White/European vs. Black) factorial design, with each participant observing one game of Cyberball in which either a White or Black target was either included or severely ostracized by other White individuals.

#### Measures

Participants reported their current feelings of guilt (*guilty*, *apologetic*, *ashamed*; *α* = .79), anger (*angry*), sadness (*sad*), and positive affect (*happy*, *comfortable*, *lively*, *calm*, *caring*; *α* = .79) on a 9-point scale (1 = *definitely do not feel*, 9 = *definitely do feel*). These items were adapted from the Brief Mood Introspection Scale (BMIS [68]) as well as the revised Positive and Negative Affect Schedule (PANAS-X [69]). In all studies where a particular emotion was assessed through multiple items, we formed an overall index of that emotion by averaging across participants’ ratings of the associated items.

Participants’ feelings of empathy toward the target were assessed with a single item embedded among the impression dimensions (see below). This item asked participants to indicate the extent to which they empathized with each player during the game of Cyberball on a 7-point scale (1 = *not at all*, 7 = *very much*).

Participants indicated their impressions of all the players in Cyberball, including the target, along several dimensions. Ratings were made separately for each player with the order of the players randomized across participants. Participants indicated how *likeable* they thought each player was and how much they would *trust* him or her, as well as how much they *identified* with and felt *similar* to each player. Ratings were made on a 7-point scale with higher scores indicating more favorable impressions (e.g., more likeable). In all studies where impressions were assessed with more than two items, a principal component analysis indicated that a single component accounted for most of the variance in participants’ impressions. Consequently, participants’ ratings of liking, trust, identification, and similarity were averaged together to create a single impressions of target index (*α* = .72). The focal measures employed across all studies are summarized in Table 2. Participants completed the impression items first, then the empathy measure, and then the affect items.

**Table 2:** Main dependent measures.

	Items comprising measure					
Measure	Study 1	Study 2	Study 3	Study 4	Scale	Reliability
Affective reactions						
Guilt	Guilty, apologetic, ashamed	See Study 1	Guilty, ashamed	Guilty, ashamed, blameworthy, angry, disgusted, dissatisfied with/at self	9-point (Studies 1 and 2) 5-point (Studies 3 and 4)	.79 to .89
Anger	Angry	See Study 1	Hostile, irritable	Angry, annoyed, hostile, indignant, resentful, outraged		.68 to .88
Sadness	Sad	See Study 1	Distressed, upset	Sad, blue, downhearted, alone, lonely		.78 to .87
Fear	--	--	Scared, nervous, jittery, afraid	Scared, nervous, jittery, afraid, frightened, shaky		.83 to .89
Positive affect	Happy, comfortable, lively, calm, caring	See Study 1	Excited, enthusiastic, interested, proud, strong, alert, inspired, determined, attentive, active	Happy, joyful, delighted, cheerful, excited, enthusiastic, lively, energetic, proud, strong, confident, bold, daring, fearless		.79 to .95
Behavioral responses						
Messages sent	--	--	--	Sending messages to target or perpetrators	--	--
Message valence				How supportive/angry messages were	7-point	.82 to .95
Partner choice	--	--	--	Choice of target or perpetrator for partner	--	--
Feelings of empathy						
Empathy toward target	Empathized with	———————See Study 1——————--		Moved, compassionate, warm, soft-hearted, sympathetic, tender	7-point	.88
Positivity of impressions of target						
Impressions of target	Liking, trust, identification, similarity	———————See Study 1——————--		Identification, similarity	7-point	.72 to .91

Reliability coefficients for each measure are Cronbach’s alpha except for message valence which are intraclass correlations. For all variables larger scale numbers indicate greater endorsement or more of each construct.

## Study 2

### Method

#### Participants

A sensitivity power analysis indicated that the sample size of 924 would provide .80 power to detect an effect of *f* = 0.102 ($\eta_{p}^{2}$ = .007).

#### Procedure and design

The procedure and design was the same as Study 1 except for the addition of the moderate ostracism condition.

#### Measures

Participants’ feelings of guilt (*α* = .79), anger, sadness, positive affect (*α* = .82), empathy toward the target, and impressions of the target (*α* = .85) were all assessed in the same way as Study 1.

## Study 3

### Method

#### Participants

A sensitivity power analysis indicated that the sample size of 1958 would provide .80 power to detect an effect of *f* = 0.070 ($\eta_{p}^{2}$ = .003).

#### Procedure and design

The procedure and design of Study 3 was the same as in Study 2 except that photos of men in addition to women were employed in Cyberball.

#### Measures

Participants reported their present feelings of guilt (*guilt*, *ashamed*; *α* = .80), anger (*hostile*, *irritable*; *α* = .68), sadness (*distressed*, *upset*; *α* = .78), fear (*scared*, *nervous*, *jittery*, *afraid*; *α* = .83), and positive affect (*excited*, *enthusiastic*, *interested*, *proud*, *strong*, *alert*, *inspired*, *determined*, *attentive*, *active*; *α* = .91) on a 5-point scale (1 = *very slightly or not at all*, 5 = *extremely*). These items were adapted from the Positive and Negative Affect Schedule (PANAS [70]). The order of the items was randomized. Participants’ feelings of empathy toward the target as well as their impressions of the target (*α* = .86) were measured in the same way as in Studies 1 and 2.

## Study 4

### Method

#### Participants

A sensitivity power analysis indicated that the sample size of 1230 would provide .80 power to detect an effect of *f* = 0.080 ($\eta_{p}^{2}$ = .003; Odds Ratio [OR] = 1.34).

#### Procedure and design

The procedure and design of Study 4 was the same as Study 1, except for the addition of male photos in Cyberball.

#### Measures

Participants reported their present feelings of guilt (*guilty*, *ashamed*, *blameworthy*, *angry at self*, *disgusted with self*, *dissatisfied with self*; *α* = .89), anger (*angry*, *annoyed*, *hostile*, *indignant*, *resentful*, *outraged*; *α* = .88), sadness (*sad*, *blue*, *downhearted*, *alone*, *lonely*; *α* = .87), fear (*scared*, *nervous*, *jittery*, *afraid*, *frightened*, *shaky*; *α* = .89), and positive affect (*happy*, *joyful*, *delighted*, *cheerful*, *excited*, *enthusiastic*, *lively*, *energetic*, *proud*, *strong*, *confident*, *bold*, *daring*, *fearless*; *α* = .95) on a 5-point scale (1 = *very slightly or not at all*, 5 = *extremely*). These items were adapted from the PANAS-X [69] as well as past research in the intergroup domain [52]. The order of the items was randomized.

Participants’ feelings of empathy toward the target were measured by asking participants to indicate the degree to which they currently felt *moved*, *compassionate*, *warm*, *soft-hearted*, *sympathetic*, and *tender*. These items were adapted from the empathy literature [71,72] and were included among the other affect items and rated on the same scale. Participants’ ratings across the items were averaged together (*α* = .88).

Participants’ impressions of the target were assessed in terms of identification and similarity only. Because there were only two items we did not perform a PCA on impressions of the target in Study 4. These items were highly correlated and so were averaged together to create an impressions of target index (*r* = .84; *α* = .91).

In Study 4 we also measured a number of distinct behavioral responses to the witnessed interaction. First, we evaluated whether participants acted to try to alleviate any distress the target may have been experiencing by giving participants the opportunity to select him or her as a partner for a second, ostensible task. That is, participants were able to directly reinclude the ostracized target. Participants were told that there was a short, additional final task for them to complete with another participant. They were asked to select a partner for the task from the three individuals who had previously took part in Cyberball. Participants were further informed that a message would be sent to the individual they selected notifying them that they had been chosen. Photos identical to those shown during Cyberball were displayed to assist participants with their choice. Participants’ selection was coded dichotomously (0 = selected perpetrator; 1 = selected target).

We further assessed whether participants opted to send a message to the target or the perpetrators by giving them the option to send messages to any or none of the Cyberball players (both perpetrators separately and target). Photographs of each of the players were presented alongside a text-box in which participants could enter their message. We coded whether or not participants sent a message to the target (did not send message to target = 0; sent message to target = 1) or one of the perpetrators (did not send message to a perpetrator = 0; sent message to perpetrator = 1).

The content of participants’ open-ended messages was also rated by three coders blind to participants’ condition assignments and the study’s specific objectives. Coders rated how supportive and angry each of the messages were using a 7-point scale (1 = *not at all*, 7 = *very much*). The supportiveness of the messages was coded with the following four items: “Does it seem like they trying to comfort this person?”, “how supportive are they toward this person?”, “how much do they seem like they are trying to help this person?”, and “how much do they seem to be sympathizing with this person?”. The anger in the messages was coded with the following four items: “How upset with or angry are they toward this person?”, “does it seem like they are trying to chastise, criticize, or reprimand this person?”, “does it seem like they are blaming this person?”, “how much do they seem to be derogating this person?”. The dimensions on which coders made their ratings of participants’ messages were both adapted from past work examining responses to observed acts of ostracism [73] and supplemented with additional items we created. Coding participants’ messages in this way allowed us to examine the degree to which participants attempted to assuage the target’s distress as well as the extent to which they sought to “call out” the perpetrators’ treatment of the target and perhaps try to persuade them of the harm such actions may cause. Coders’ overall ratings of supportiveness (intraclass correlation coefficient [ICC] = .79 and .78) and anger (both ICCs = .96) for each of the perpetrators were reliable and highly correlated (*r*s = .84 and .90 for supportiveness and anger respectively) and so were averaged together to a create general index of how supportive (ICC = .92) and angry (ICC = .95) participants’ messages were to both perpetrators combined. Coders’ average ratings of the supportiveness (ICC = .82) and anger (ICC = .94) of participants’ messages to the target were also reliable. In this study participants completed the affect items first, followed by the empathy measure, impressions, partner choice, and messaging opportunity.

### Results

Because preliminary analyses of affective and behavioral reactions in Studies 3 and 4 including sex as an additional factor did not yield any consistent effects we do not discuss this factor further (for the results of these analyses see the supporting information, S1 File). Unless otherwise indicated the effects of observed treatment and target type on all dependent variables were examined through factorial analysis of variance (ANOVA). The results of omnibus hypothesis tests for affective reactions, empathy, impressions, and behavioral responses are summarized in Tables 3 and 4. The results of simple effect tests examining the effect of target type within each of the observed treatment conditions are presented in Table 5 for variables where significant interactions emerged.

**Table 3:** Results of omnibus hypothesis tests for affective reactions, empathy, and impressions in Study 4.

Measure	Effect	*df*	*F*	*p*	$\eta_{p}^{2}$ 90% CI
Affective reactions					
Anger	OT	1,1225	66.93	< .001	.052 [.033, .073]
	TT	1,1225	3.20	.074	.003 [.000, .010]
	OT × TT	1,1225	23.84	< .001	.019 [.008, .034]
Guilt	OT	1,1225	0.07	.786	.000 [.000, .002]
	TT	1,1225	0.03	.866	.000 [.000, .001]
	OT × TT	1,1225	3.56	.059	.003 [.000, .010]
Sadness	OT	1,1226	8.58	.003	.007 [.001, .017]
	TT	1,1226	0.20	.652	.000 [.000, .003]
	OT × TT	1,1226	3.24	.072	.003 [.000, .010]
Fear	OT	1,1225	0.00	.974	.000 [.000, .000]
	TT	1,1225	0.21	.651	.000 [.000, .003]
	OT × TT	1,1225	4.68	.031	.004 [.000, .012]
Positive affect	OT	1,1226	12.00	.001	.010 [.003, .021]
	TT	1,1226	0.00	.959	.000 [.000, .000]
	OT × TT	1,1226	0.22	.643	.000 [.000, .003]
Feelings of empathy					
Empathy	OT	1,1225	20.84	< .001	.017 [.007, .031]
	TT	1,1225	2.04	.154	.002 [.000, .008]
	OT × TT	1,1225	0.50	.482	.000 [.000, .004]
Positivity of impressions of target					
Impressions	OT	1,1226	20.57	< .001	.016 [.007, .030]
	TT	1,1226	11.28	< .001	.009 [.002, .020]
	OT × TT	1,1226	1.17	.280	.001 [.000, .006]

OT = Observed Treatment; TT = Target Type; CI = confidence interval. 90% confidence intervals around partial η^2^ squared are equivalent to 95% confidence intervals around *d* [74].

**Table 4:** Results of omnibus hypothesis tests for behavioral responses in Study 4.

Measure	Effect	*df*	*F*	*p*	$\eta_{p}^{2}$ 90% CI
Supportiveness and anger of messages sent to target					
Supportiveness	OT	1,484	135.76	< .001	.219 [.168, .270]
	TT	1,484	0.96	.328	.002 [.000, .014]
	OT × TT	1,484	0.03	.871	.000 [.000, .003]
Anger	OT	1,484	1.34	.248	.003 [.000, .016]
	TT	1,484	0.93	.335	.002 [.000, .014]
	OT × TT	1,484	0.57	.452	.001 [.000, .012]
Supportiveness and anger of messages sent to perpetrators					
Supportiveness	OT	1,475	247.37	< .001	.342 [.288, .393]
	TT	1,475	0.09	.763	.000 [.000, .007]
	OT × TT	1,475	0.02	.881	.000 [.000, .002]
Anger	OT	1,475	373.38	< .001	.440 [.388, .486]
	TT	1,475	0.05	.831	.000 [.000, .004]
	OT × TT	1,475	0.05	.821	.000 [.000, .004]
Decision to send message to target or perpetrator					
	Effect	*β* (*SE*)	Wald	*p*	*OR* 95% CI
Sent to target	OT	0.48 (.12)	16.79	< .001	1.62 [1.29, 2.04]
	TT	0.10 (.12)	0.66	.415	1.10 [0.87, 1.39]
	OT × TT	0.31 (.24)	1.77	.183	1.37 [0.86, 2.17]
Sent to perp	OT	0.37 (.12)	9.70	.002	1.44 [1.15, 1.82]
	TT	0.12 (.12)	1.04	.308	1.13 [0.90, 1.42]
	OT × TT	0.36 (.24)	2.36	.124	1.44 [0.91, 2.28]
Choice of partner for final task					
Partner choice	OT	2.03 (.14)	223.92	< .001	7.58 [5.81, 9.88]
	TT	-0.11 (.13)	0.71	.400	0.90 [0.69, 1.16]
	OT × TT	0.04 (.27)	0.03	.874	1.04 [0.61, 1.78]

OT = Observed Treatment; TT = Target Type; CI = confidence interval; perp = perpetrator; OR = odds ratio. 90% confidence intervals around partial η^2^ squared are equivalent to 95% confidence intervals around *d* [74].

**Table 5:** Results of hypothesis tests of the simple effect of target type in Study 4.

Measure	Condition	*df*	*F*	*p*	$\eta_{p}^{2}$ 90% CI	Direction of effect
Anger	SvOstracism	1,1225	22.01	< .001	.018 [.008, .032]	BLK > WHT
	Inclusion	1,1225	4.84	.028	.004 [.000, .012]	BLK < WHT
Guilt	SvOstracism	1,1225	1.46	.227	.001 [.000, .007]	BLK > WHT
	Inclusion	1,1225	2.14	.144	.002 [.000, .008]	BLK < WHT
Sadness	SvOstracism	1,1226	2.51	.114	.002 [.000, .008]	BLK > WHT
	Inclusion	1,1226	0.92	.338	.001 [.000, .006]	BLK < WHT
Fear	SvOstracism	1,1225	1.45	.229	.001 [.000, .007]	BLK > WHT
	Inclusion	1,1225	3.46	.063	.003 [.000, .001]	BLK < WHT

SvOstracism = Severe Ostracism Condition; CI = confidence interval; BLK = black target; WHT = White/European target. 90% confidence intervals around partial η^2^ squared are equivalent to 95% confidence intervals around *d* [74].

#### Affective reactions

A significant Observed Treatment **×** Target Type interaction emerged for feelings of anger. After observing severe ostracism, participants felt more anger when the target was Black (*M* = 1.78, *SD* = 0.85) as opposed to White (*M* = 1.52, *SD* = 0.68), whereas after observing inclusion, they felt less anger when the target was Black (*M* = 1.27, *SD* = 0.51), as opposed to White (*M* = 1.39, *SD* = 0.62). A significant main effect of observed treatment and a marginally significant main effect of target group membership, both qualified by the aforementioned interaction, were also evident for anger.

The Observed Treatment **×** Target Type interaction was marginally significant for guilt. Although descriptively the means followed the same pattern as for anger, none of the simple effects of target type were significant in either observed treatment condition.

A marginally significant Observed Treatment **×** Target Type interaction was also evident for sadness. Although descriptively the means followed the same pattern as for anger, the simple effect of target group membership was not significant in either observed treatment condition. A significant main effect of observed treatment also emerged, such that after observing severe ostracism (*M* = 1.68, *SD* = 0.82) relative to inclusion (*M* = 1.55, *SD* = 0.78), participants felt more sadness.

A significant Observed Treatment **×** Target Type interaction also emerged for fear. The simple effect of target group membership was only marginally significant in the inclusion condition, such that participants experienced more fear when the target was White (*M* = 1.32, *SD* = 0.60) relative to Black (*M* = 1.24, *SD* = 0.52).

For participants’ positive affect a significant main effect of observed treatment was evident, such that after observing severe ostracism (*M* = 1.89, *SD* = 0.82) relative to inclusion (*M* = 2.07, *SD* = 0.92), participants felt less positive affect.

#### Feelings of empathy toward target

A significant main effect of observed treatment emerged on participants’ feelings of empathy toward the target, with participants empathizing more with severely ostracized (*M* = 2.25, *SD* = 0.90) as opposed to included targets (*M* = 2.01, *SD* = 0.93).

#### Impressions of target

A significant main effect of observed treatment was evident on participants’ overall impressions of the target, such that participants reported more positive impressions of severely ostracized (*M* = 3.76, *SD* = 1.51) relative to included targets (*M* = 3.38, *SD* = 1.43). A significant main effect of target type also emerged, with participants reporting less positive impressions of Black (*M* = 3.43, *SD* = 1.44) compared to White targets (*M* = 3.71, *SD* = 1.51).

#### Behavioral responses

We employed hierarchical logistic regression to determine whether participants’ choice of partner (0 = selected perpetrator; 1 = selected target) for the ostensible final task was impacted by observed treatment (inclusion = 0; severe ostracism = 1) and target type (White/European = 0, Black = 1). We entered observed treatment and target type in Step 1 and added their interaction in Step 2. A significant main effect of observed treatment emerged, such that after observing severe ostracism, relative to observing inclusion, participants were over 7 times more likely to select the target as opposed to one of the perpetrators as their partner for the upcoming task.

With respect to the messaging dependent measure, a significant main effect of observed treatment on the supportiveness of participants’ messages to the target was evident, such that participants sent more positive, supportive messages to the target after they observed him or her being severely ostracized (*M* = 3.54, *SD* = 1.22) than after observing him or her being included (*M* = 2.40, *SD* = 0.82). Descriptive statistics for all behavioral responses are reported in Table 6.

**Table 6:** Descriptive statistics for behavioral responses in Study 4.

Measure	White/European target				Black target					
	Inclusion		Severe ostracism		Inclusion		Severe ostracism			
	Supportiveness and anger of messages sent to target									
									Range	
	*M*	*SD*	*M*	*SD*	*M*	*SD*	*M*	*SD*	Potential	Actual
Supportiveness	2.35	0.9	3.5	1.22	2.46	0.72	3.58	1.22	7-Jan	1.0–6.50
Anger	1.34	0.82	1.22	0.54	1.23	0.73	1.21	0.61	7-Jan	1.0–6.42
	Supportiveness and anger of messages sent to perpetrators									
Supportiveness	2.38	0.83	1.4	0.51	2.36	0.78	1.39	0.58	7-Jan	1.0–4.46
Anger	1.31	0.67	3.28	1.1	1.31	0.78	3.32	1.52	7-Jan	1.0–7.0
	Decision to send message to target or perpetrator									
	Count	*%*	count	%	count	%	count	%	Total count	Total %
Sent to target	108	34.73	124	42.32	103	33.23	153	48.42	488	39.67
Sent to perp	110	35.37	116	35.59	105	33.87	148	46.84	479	38.94
*N*	311		293		310		316		1230	
	Choice of partner for final task									
Selected target	124	40	242	83.16	114	37.01	259	81.96	739	60.33
*N*	310		291		308		316		1225	

Examples of supportive messages to the target included: “I don't know what's up with your exclusion from the game but, I disagree with it.” (supportiveness = 3.58) and “Hey there! You did good! but for some reason player 1 and player 3 didn't want to play with you for the rest of the game huh” (supportiveness = 3.58). However, as noted in Table 6, there was a wide range, with some participants’ messages indicating complete unconcern for the target’s mistreatment “Hi there” (supportiveness = 1.50) and others, such as that presented below, a high degree of concern:

“Glad you stuck around while those other two players, those IDIOTS, hogged the ball and played catch solely with each other. Don't let it get to you. It doesn't have anything to do with you, the one person in the group I'd LIKE to play ball with, it has to do with them, their thoughtlessness, their selfishness, etc. Somehow, I think you know all this already. I think you're the only one with any brains or any heart in the group.” (supportiveness = 5.92).

For participants’ messages to the perpetrators, a significant main effect of observed treatment emerged for both how angry and how supportive the messages were, such that participants sent more angry messages to the perpetrators after observing them engage in severe ostracism (*M* = 3.30, *SD* = 1.35) than after observing them engage in inclusion (*M* = 1.31 *SD* = 0.73), whereas they sent more supportive messages after observing them include (*M* = 2.37, *SD* = 0.80) as opposed to severely ostracize the target (*M* = 1.39, *SD* = 0.55).

Examples of angry messages to the perpetrators included: “Let player two participate more.” (anger = 3.33) and “Why would you all of a sudden avoid Player 2?” (anger = 4.00). Yet, there was a wide range (see Table 6), with some participants’ messages not revealing any anger toward the perpetrators, “throw in a triangle then reverse” (anger = 1.96), and other messages a high degree of anger, “Why did you feel the need to be an asshole and not pass it to player 2?” (anger = 6.29).

We conducted a hierarchical logistic regression parallel to the one for partner choice to examine the effect of observed treatment and target type on participants’ decision to send a message to the target or perpetrators. This analysis yielded a significant main effect of observed treatment, with participants being over 1.5 times more likely to send targets a message after observing them be severely ostracized as opposed to included. For participants’ decision to send a message to one of the perpetrators, a significant main effect of observed treatment was evident, with participants being almost 1.5 times more likely to send a message to one of the perpetrators after observing them severely ostracize, relative to include, the target.

#### Mediation analyses

Although there were significant interactions between observed treatment and target type on feelings of anger and on feelings of fear, there were no parallel direct effects on behavior. To assess the possibility that observing ostracism had indirect effects on behavior via affective reactions [75] we used the PROCESS macro v2.13 (Model 8, with 10,000 bootstrap samples [76]) to test for indirect behavioral effects via anger and fear on participants’ choice of partner, their decision to send a message to the target or one of the perpetrators and the supportiveness and anger of those messages. For all analyses, target type (White/European = 0; Black = 1) served as the predictor and observed treatment (inclusion = 0; severe ostracism = 1) functioned as the moderator.

No significant indirect effects via fear emerged (all 95% bootstrap CIs for the indirect effects included zero for all measures).

Significant indirect effects via anger were present on all measures except anger of participants’ messages to the target. The indirect effects are summarized in Table 7. As participants felt significantly more anger after witnessing the severe ostracism of a Black as opposed to a White target, these results suggest that observing the severe ostracism of a disadvantaged ethnic minority group member had the indirect effect, via increased anger, of increasing participants’ choice of the Black target as their partner for a future task, their likelihood of sending the Black target a message that was more likely to be positive and encouraging, as well as their likelihood of sending one of the perpetrators a message that was more likely to be negative (i.e., angry, chastising) and less positive (i.e., supportive, helpful). Several indirect effects also emerged in the inclusion condition (see Table 7). Because participants felt significantly less anger after witnessing the inclusion of a Black as opposed to a White target, these indirect effects suggest that observing inclusion of a disadvantaged group member had the indirect effect, via reduced anger, of decreasing participants’ choice of the Black target as a partner in a future task and their likelihood of sending a message to the perpetrators or target. Thus, it was only after observing negative treatment in the form of ostracism that increased feelings of anger appeared to motivate observers’ efforts to not only comfort and support disadvantaged group targets, but also confront and challenge the perpetrators of such mistreatment.

**Table 7:** Indirect effect of target type via anger on all behavioral responses in Study 4.

	Indirect effect: *b* and 95% CI	
Measure/condition	Inclusion	Severe ostracism
Choice of partner	-0.04* [-.0900, -.0067]	0.08* [.0272, .1666]
Perpetrator oriented measures		
Likelihood of sending message to perpetrators	-0.04* [-.0834, -.0089]	0.09* [.0344, .1551]
Supportiveness of messages to perpetrators	0.02 [-.0043, .0384]	-0.03* [-.0671, -.0062]
Anger of messages to perpetrators	-0.05 [-.1161, .0133]	0.10* [.0254, .2022]
	Target oriented measures	
Likelihood of sending message to target	-0.04* [-.0858, -.0098]	0.09* [.0376, .1605]
Supportiveness of messages to target	-0.02 [-.0654, .0114]	0.06* [.0154, .1331]
Anger of messages to target	0.0003 [-.0088, .0101]	-0.001 [-.0208, .0213]

Participants felt more anger after witnessing the severe ostracism of a Black as opposed to a White/European target, but less anger after observing the inclusion of a Black relative to a White/European target. Indirect effects were computed using the PROCESS macro v2.13, Model 8, with 10,000 bootstrap samples [76]. Predictor = target type (White/European = 0; Black = 1); moderator = observed treatment (inclusion = 0; severe ostracism = 1); CI = confidence interval.

* *p* < .05

## Meta-analysis of Studies 1 to 4

We now report the meta-analysis of all four studies. We first outline the approach that we employed to create a combined summary of the results of the individual studies. We then summarize the results of this analysis.

### Meta-analytic approach

To meta-analyze results across studies we computed an estimate of the average standardized mean difference in reactions to observing a disadvantaged group target relative to a dominant group target in the form of Cohen’s *d* across all studies in each observed treatment condition. The weights used to pool individual effect sizes were calculated using the inverse variance method. Given that the studies we conducted examined similar hypotheses using highly similar procedures and measures, we report the average effect sizes computed using a fixed-effects model. The average effect size for each dependent variable that appeared in two or more studies for each condition are presented in Table 8.

**Table 8:** Overall effect of target type for affective reactions, empathy toward target, and impressions of target in each observed treatment condition.

Measure/condition	Inclusion (Studies 1 to 4)			Moderate ostracism (Studies 2 and 3 only)			Severe ostracism (Studies 1 to 4)		
	*d* 95% CI	*Q*	*I*^2^	*d* 95% CI	*Q*	*I*^2^	*d* 95% CI	*Q*	*I*^2^
Affective reactions									
Guilt	-0.06 [-0.15, 0.04]	0.99	0.00	**0.11†** **[-0.02, 0.23]**	0.75	0.00	0.18*** **[0.08, 0.27]**	1.60	0.00
Anger	-0.06 [-0.16, 0.03]	6.95**†**	56.86	0.10 [-0.02, 0.23]	9.22**	89.19	0.17*** **[0.07, 0.26]**	8.08*	62.88
Sadness	-0.07 [-0.16, 0.03]	2.43	0.00	0.08 [-0.05, 0.21]	2.71**†**	63.10	0.13** **[0.03, 0.22]**	0.58	0.00
Fear	-0.05 [-0.16, 0.06]	2.78**†**	64.05	-0.10^b^ [-0.25, 0.05]	--	--	0.08 [-0.04, 0.19]	0.13	0.00
Positive affect	-0.02 [-0.11, 0.08]	1.27	0.00	-0.02 [-0.15, 0.10]	0.08	0.00	-0.02 [-0.11, 0.08]	0.88	0.00
Feelings of empathy									
Empathy toward target	**0.08†** **[-0.01, 0.18]**	4.43	32.23	0.14* **[0.01, 0.26]**	5.73*	82.55	0.02 [-0.07, 0.12]	4.09	26.73
Positivity of impressions of target									
Impressions of target	0.03 [-0.07, 0.12]	12.60**	76.19	0.15* **[0.03, 0.28]**	2.37	57.86	-0.13** **[-0.22, -0.03]**	4.51	33.48

Positive Cohen’s *d*s indicate greater reactivity toward Black targets (i.e., higher mean scores on a dependent measure when target was a Black individual), whereas negative Cohen’s *d*s indicate greater reactivity to White/European targets. Cohen’s *d* was computed using a fixed-effects model. CI = confidence interval; *Q* = Cochran’s *Q* and *I*^2^ = *I*^2^ index used to test for and quantify the degree of heterogeneity in effect sizes, respectively.

^b^ = Cohen’s *d* based on a single study.

† *p* < .10

* *p* < .05

** *p* < .01

*** *p* < .001

Alongside estimates of the average effect size we also evaluated the degree of heterogeneity in effect sizes across studies that is not due to sampling error. We evaluated heterogeneity using both Cochran’s *Q* and the *I*^2^ index [77]. Cochran’s *Q* assesses whether there is significant heterogeneity in effect sizes, with a significant *Q* indicating heterogeneity. The *I*^2^ index, on the other hand, provides a percentage estimate of the extent of heterogeneity. The *I*^2^ index ranges from 0% to 100%, with values of 25%, 50%, and 75% indicating low, moderate, and high heterogeneity. Heterogeneity information accompanies all effect size estimates.

To determine whether the overall effect of target type differed as a function of observed treatment we conducted moderator analyses [20,78]. The overall effect of target type across all observed treatment conditions, the omnibus test of moderation, and the outcome of contrasts comparing each of the specific observed treatment conditions are presented in Table 9.

**Table 9:** Overall effect of target type and tests of moderation for affective reactions, empathy toward target, and impressions of target.

					Contrast					
Measure	Effect across conditions			Omnibus moderation	moderate vs. inclusion		severe vs. inclusion		severe vs. moderate	
	*d* 95% CI	*Q*	*I*^2^	*QM*(*df*)	*B*	*SE*	*B*	*SE*	*B*	*SE*
Affective reactions										
Guilt	0.07* **[0.01, 0.13]**	15.64†	42.47	12.30(2)**	0.17*	0.08	0.24***	0.07	0.07	0.08
Anger	0.06* **[0.003, 0.12]**	36.10***	75.07	11.85(2)**	0.17*	0.08	0.23***	0.07	0.06	0.08
Sadness	0.04 [-0.02, 0.10]	14.37	37.35	8.65(2)*	**0.15**†	0.08	0.20**	0.07	0.05	0.08
Fear	**-**0.01 [-0.08, 0.06]	7.18	44.30	4.27(2)	-0.05	0.10	0.13	0.08	**0.18**†	0.10
Positive affect	**-**0.02 [-0.08, 0.04]	2.24	0.00	0.004(2)	-0.003	0.08	0.002	0.07	0.01	0.08
Feelings of empathy										
Empathy toward target	0.07* **[0.01, 0.13]**	16.44†	45.24	2.18(2)	0.05	0.08	-0.06	0.07	-0.12	0.08
Positivity of impressions of target										
Impressions of target	**-**0.006 [-0.06, 0.05]	32.24***	72.08	12.75(2)**	0.13	0.08	-0.15*	0.07	-0.28***	0.08

Positive Cohen’s *d*s indicate greater reactivity toward Black targets (i.e., higher mean scores on a dependent measure when target was a Black individual), whereas negative Cohen’s *d*s indicate greater reactivity to White/European targets. Cohen’s *d* was computed using a fixed-effects model. CI = confidence interval; *Q* = Cochran’s *Q* and *I*^2^ = *I*^2^ index used to test for and quantify the degree of heterogeneity in effect sizes, respectively; *QM* = omnibus test of moderator model coefficients; *df* = degrees of freedom; *SE* = standard error.

† *p* < .10

* *p* < .05

** *p* < .01

*** *p* < .001

We also computed the average effect of observed treatment for each dependent variable separately for Black and White targets as well as tested whether the overall effect differed as a function of target type. For these analyses we averaged the moderate and severe ostracism conditions within Studies 2 and 3 before computing the average effect to avoid introducing a dependency in the data by utilizing the inclusion condition twice in effect size computations [78]. The results of these analyses are presented in Tables 10 and 11. Notably, parsing the interaction between observed treatment and target type in this way leads to the same conclusions as focusing on the effect of target type. Consequently, to keep the presentation of results manageable we focus our discussion on the effect of target type. All meta-analytic analyses were conducted using the *metafor* [79] and *meta* [80] packages in the R statistical environment [81].

**Table 10:** Overall effect of observed treatment for affective reactions, empathy toward target, and impressions of target for each target type.

Measure/condition	White/European target			Black target		
	*d* 95% CI	*Q*	*I*^2^	*d* 95% CI	*Q*	*I*^2^
Affective reactions						
Guilt	0.07 [-0.01, 0.16]	20.02***	85.02	0.27*** **[0.18, 0.36]**	25.93***	88.43
Anger	0.30*** **[0.22, 0.39]**	16.45***	81.77	0.51*** **[0.42, 0.60]**	30.81***	90.26
Sadness	0.25*** **[0.16, 0.34]**	8.13*	63.09	0.42*** **[0.33, 0.50]**	19.54***	84.65
Fear	-0.04 [-0.14, 0.06]	1.76	43.04	0.03 [-0.07, 0.13]	2.63	61.99
Positive affect	-0.17*** **[-0.26, -0.08]**	26.31***	88. 60	-0.18** [-0.26, -0.09*]	16.80***	82.14
Feelings of empathy						
Empathy toward target	0.50*** **[0.42, 0.59]**	21.44***	86.01	0.47*** **[0.39, 0.56]**	8.92*	66.35
Positivity of impressions of target						
Impressions of target	0.12** **[0.03, 0.21]**	11.47**	73.85	0.02 [-0.07, 0.10]	9.47*	68.31

Positive Cohen’s *d*s indicate greater reactivity to ostracism (i.e., higher mean scores on a dependent measure when the target was ostracized), whereas negative Cohen’s *d*s indicate greater reactivity to inclusion. Cohen’s *d* was computed using a fixed-effects model. The overall effect of observed treatment collapses across the moderate and severe ostracism conditions. CI = confidence interval; *Q* = Cochran’s *Q* and *I*^2^ = *I*^2^ index used to test for and quantify the degree of heterogeneity in effect sizes, respectively.

* *p* < .05

** *p* < .01

*** *p* < .001

**Table 11:** Overall effect of observed treatment and tests of moderation for affective reactions, empathy toward target, and impressions of target.

					Contrast	
Measure	Effect across target type			Omnibus moderation	Black vs. White/European target	
	*d* 95% CI	*Q*	*I*^2^	*QM*(*df*)	*B*	*SE*
Affective reactions						
Guilt	0.17*** **[0.11, 0.23]**	55.84***	87.47	9.89(1)**	0.20**	0.06
Anger	0.40*** **[0.34, 0.47]**	57.45***	87.82	10.18(1)**	0.20**	0.06
Sadness	0.33*** **[0.27, 0.39]**	34.81***	79.89	7.14(1)**	0.17**	0.06
Fear	**-**0.01 [-0.08, 0.07]	5.17	41.94	0.78(1)	0.06	0.07
Positive affect	-0.17*** **[-0.24, -0.11]**	43.12***	83.77	0.01(1)	-0.01	0.06
Feelings of empathy						
Empathy toward target	0.49*** **[0.43, 0.55]**	30.58***	77.11	0.22(1)	-0.03	0.06
Positivity of impressions of target						
Impressions of target	0.07* **[0.01, 0.13]**	23.56**	70.29	2.63(1)	-0.10	0.06

Positive Cohen’s *d*s indicate greater reactivity to ostracism (i.e., higher mean scores on a dependent measure when the target was ostracized), whereas negative Cohen’s *d*s indicate greater reactivity to inclusion. Cohen’s *d* was computed using a fixed-effects model. The overall effect of observed treatment collapses across the moderate and severe ostracism conditions CI = confidence interval; *Q* = Cochran’s *Q* and *I*^2^ = *I*^2^ index used to test for and quantify the degree of heterogeneity in effect sizes, respectively; *QM* = omnibus test of moderator model coefficients; *df* = degrees of freedom; *SE* = standard error.

* *p* < .05

** *p* < .01

*** *p* < .001

### Meta-analytic results

#### Affective reactions

Tests of moderation revealed that the combined effect of target type on guilt, anger, and sadness was not consistent across observed treatment conditions (see Table 9). In each case the severe ostracism condition significantly differed from the inclusion condition. Indeed, in the severe ostracism condition participants reported significantly more feelings of guilt, anger, and sadness after observing an interaction with a Black as opposed to White target, whereas in the inclusion condition the opposite tended to be descriptively true, although not statistically significant (see Table 8 for the within-condition simple effects). These effects were small in magnitude. Further, for guilt and anger, the effect of target type in the moderate ostracism condition also significantly differed from its effect in the inclusion condition, whereas for sadness this difference only approached statistical significance. However, although the effect of target type on guilt, anger, and sadness in the moderate ostracism condition descriptively mirrored that obtained in the severe ostracism condition it was not statistically significant.

Overall, across observed treatment conditions participants reported significantly more feelings of guilt and anger after observing an interaction involving a Black relative to a White target, although these main effects were qualified by the aforementioned interactions. The main effect of target type was not significant for sadness. No significant effects emerged for fear or positive affect.

#### Feelings of empathy toward target

Tests of moderation indicated that the combined effect of target type on participants’ feelings of empathy toward the target was relatively consistent across observed treatment conditions. Overall, across studies, participants reported significantly greater feelings of empathy toward a Black compared to a White target. The size of this effect was small.

#### Impressions of target

Tests of moderation suggested that the influence of target type diverged across observed treatment conditions. The severe ostracism condition significantly differed from both the inclusion and moderate ostracism conditions. In the severe ostracism condition participants reported significantly more negative impressions of Black relative to White targets, whereas in the moderate ostracism and inclusion conditions the effect of target type was reversed, although it was not statistically significant in the inclusion condition. The effect of target type did not differ between the inclusion and moderate ostracism conditions: Only comparisons with severe ostracism yielded significant results.

## Discussion

Across four studies involving over 4000 participants from university and community samples we found that after observing a severe act of ostracism perpetrated by fellow dominant group members, White individuals reported significantly more feelings of guilt, anger, and sadness when the target was an ethnic minority as opposed to a White person. These results directly build on research demonstrating that individuals are affected by merely observing another person being ostracized (i.e., “vicarious ostracism”) by highlighting that individuals’ affective reactions to observing ostracism perpetrated by their fellow dominant group members vary according to whether the target is a member of a dominant or disadvantaged ethnic group. As previous work has not directly examined the effect of the target’s ethnic group membership on observers’ reactions, our findings offer novel insights into the factors that moderate how individuals respond to witnessing the ostracism of others.

Overall, the present results are not consistent with the possibility that dominant group members, as a result of an empathy gap [23], bystander apathy [36,37], or motivation to maintain a positive social identity [31], will be less impacted by witnessing ingroup members ostracize a disadvantaged group member as compared to a fellow dominant group member (*reduced reactivity hypothesis*). In fact, results of the meta-analysis conducted across the four studies indicated that in no condition were participants’ affective reactions significantly weaker when the target was a disadvantaged ethnic minority group member as opposed to a dominant group member. Further, we found little evidence of different levels of empathy for disadvantaged as compared to dominant targets of severe ostracism. For other affective reactions, such as anger, sadness, and guilt, when participants’ reactions across studies were combined together the results indicate that observers’ reactions to dominant-on-disadvantaged ostracism was more negative than reactions to dominant-on-dominant ostracism (*enhanced reactivity hypothesis)*.

Why were observers more affected by dominant-on-disadvantaged ostracism? A key possibility is that individuals found such ostracism more grievous than dominant-on-dominant ostracism because it seemed to reflect discrimination based on the target’s ethnicity. The violation of contemporary norms condemning prejudice and discrimination may have engendered anger and sadness [45]. Moreover, as a result of the salience of their shared dominant group membership with the perpetrators, observers may have subsequently felt guilty by virtue of a social identity threat produced by the perception that members of their ingroup had behaved in a racist manner.

An alternative possibility is that participants’ more negative reactions to the ostracism of a disadvantaged ethnic minority group member merely reflect what they think is the socially expected or acceptable response. However, for several reasons we consider this an unlikely explanation for our findings. Two key considerations are that participants completed all surveys anonymously online (and were reminded of the anonymity and confidentiality of their responses before taking part), and the manipulations were between-subjects such that participants were not aware of the comparison conditions. Another is that we did not include any explicit mention of ethnicity until the very end of the survey, after all the dependent measures had been collected. Both of these aspects of our methodology should have reduced social desirability concerns. Moreover, the focal pattern of results was essentially unchanged when we excluded participants who were suspicious that the purpose of the study was to examine racism and discrimination (see S1 File).

In a further effort to address the plausibility of a social desirability interpretation of our results, we also assessed whether the overall effects were changed in any way when participants’ social desirability concerns were taken into account. Specifically, we performed the meta-analysis with effect sizes from Studies 2 through 4 calculated from the estimated marginal means and their respective standard errors obtained from analyses of covariance (ANCOVA) that included participants’ scores on the impression management subscale of the Balanced Inventory of Desirable Responding Short Form (BIDR-16 [82]). Standard errors were converted to standard deviations for effect size computation. The focal pattern of results obtained across the studies was not meaningfully affected by controlling for participants’ impression management concerns. Additional details about our assessment of social desirability as well as the meta-analytic effects computed with the impression management covariate are provided in the (S1 File).

Overall, the negligible impact on the meta-analytic effects of excluding potentially suspicious participants and controlling for social desirability suggests that the pattern of results that we report is not merely an artifact of demand characteristics. Further, at the same time as we acknowledge that it is difficult to rule out completely the possibility that participants’ responses were influenced by what they considered to be socially desirable reactions, we question whether it is really appropriate to view any influence of perceived social norms in such an anonymous context as this as entirely external to or separate from the person.

It is also notable that, consistent with the possibility that individuals experienced a social identity threat in response to witnessing the potentially prejudiced behavior of fellow dominant group members, we found that White individuals derogated a Black relative to a White target of particularly severe ostracism. This finding is difficult to reconcile with a social desirability account. However, it is consistent with past work indicating that exposure to social norms condoning prejudice can increase the endorsement as well as the expression of prejudice [45,83–85]. Overall, individuals’ impressions of the severely ostracized target may have been more negative in the intergroup case because they felt motivated (because of threat) or justified (because of prevailing norms) in expressing their own prejudices [86]. Disentangling which mechanism might predominate in this context is worthy of deeper investigation in future research.

Although it may be somewhat surprising that observers were more impacted by witnessing the ostracism of a disadvantaged ethnic minority group member as opposed to fellow dominant group members given research documenting that individuals display deficits in empathizing with outgroup targets [87] and tend to overestimate their reaction to prejudiced behavior (e.g., overt racism and homophobia; [35–37]), it is important to recognize that we examined a form of negative treatment distinct from that examined in previous research, one that might tap a more universal experience. Moreover, our results are consistent with theory and research highlighting the power of contemporary norms condemning prejudice and individuals’ concerns with avoiding behaving in a prejudiced manner or appearing prejudiced to others [45,47–49], as well as theory and research on the impact of group membership on observers’ responses to ostracism that stresses that when observers witness the ostracism of a dissimilar person they are inclined to attribute the ostracism to malicious motives such as prejudice or discrimination [16]. Finally, our results are also aligned with research on group-based emotions [88] which indicates that when individuals perceive their ingroup as responsible for harming [51,89] or as unfairly advantaged over an outgroup [52] they are likely to experience negative emotions such as guilt and anger.

### Affective reactions as mediators

Although we did not find different direct behavioral implications of observing dominant-on-disadvantaged as compared to dominant-on-dominant ostracism, we did find, consistent with theories that stress that emotions are an important motivator of action [88], several indirect effects through affective reactions. These analyses revealed a role for anger as the most proximal predictor of observers’ behavior. Specifically, observers felt angrier after witnessing a severe act of ostracism perpetrated against a Black relative to a White target and feelings of anger were linked to a number of behavioral responses by observers directed toward helping and supporting the target and confronting the perpetrators. These involved directly reincluding the target by selecting him or her as a partner for a future task, sending the target comforting messages, and sending the perpetrators more negative (i.e., angry, critical) and less positive (i.e., sympathetic, helpful) messages. As indirect effects of anger on behavior were evident in only one study, however, these results should be considered tentative and additional research along these lines would be particularly valuable.

Although our mediation analyses were data-driven and exploratory, the results we obtained are in line with theory and research on group-based emotions that indicates that anger may be a particularly potent motivator of action. For example, in their theoretical integration of the effects of advantaged groups’ prosocial emotions Thomas et al. [90] suggest that anger, particularly when it is experienced by advantaged group members as a function of perceiving their ingroup as responsible for the illegitimate disadvantages faced by other groups, may often motivate behavior designed to change or regulate the advantaged group, such as confronting or protesting the actions of other ingroup members [52,89].

### Effect sizes and heterogeneity

It should be acknowledged that although we found significant differences in the levels of guilt, anger and sadness that individuals experienced upon witnessing the ostracism of disadvantaged versus dominant group targets, the size of these differences was small. However, as has been frequently argued, small effects may be meaningful and should not be reflexively trivialized [91]. In the present context, where the ostracism manipulation is quite severe and reactions to it overall fairly strong given that it involves only a briefly observed virtual interaction between strangers (Cohen’s *d* for the effect of observing ostracism relative to inclusion ranged from 0.17 to 0.49 across studies for guilt, anger, sadness, and empathy), any moderation of the effect in what is, from many perspectives, an unexpected direction, seems notable. At a minimum it would seem clear that there is no evidence for reduced reactivity in this context. It is also important to note that despite being small, the differences we found on participants’ feelings of anger to observing the ostracism of a disadvantaged relative to a dominant group target were linked to increases in behavioral responses that may have meaningful consequences for targets’ well-being as well as for the continued perpetration of ostracism and discrimination.

It is also the case that the levels of the specific types of negative affect we assessed (as reported in Study 4) were not high. Notably, mean levels of positive affect were also low, suggesting that overall participants were not feeling particularly positive or negative. It may be that the online nature of the paradigm granted participants distance from the situation that dampened their reactions. It is also possible that the general framing of the affect questions pushed toward less extreme responses. That is, participants were asked about their current mood, not specifically about how the game of Cyberball they watched made them feel. Regardless, although what is most critical from our perspective, and the focus of our analysis, is the relative difference in reactivity across conditions that vary in terms of the target's status, it is nonetheless important to appreciate that the reactions that we documented were on average not very strong.

Alongside computing estimates of the average effect size across studies, we also assessed the degree of heterogeneity in effects that was not due to sampling error. Across studies the degree of heterogeneity was relatively low (Cochran’s *Q* was frequently nonsignificant and *I*^2^ index was often 0), which would be expected based on the relatively high degree of similarity in the design and methodology of our studies. Isolated instances of significant heterogeneity occurred for positive affect and impressions of the target, both in the inclusion condition, and for empathy toward the target in the moderate ostracism condition. An exception was participants’ feelings of anger, for which Cochran’s *Q* was significant in both of the ostracism conditions, and marginally significant in the inclusion condition, and the *I*^2^ indicated a moderate to high degree of heterogeneity across the conditions (56.90% - 89.20%). As discussed in more detail below, this heterogeneity might be accounted for by methodological differences across Study 4 versus the other studies. Overall, the effect of target type on participants’ affective reactions was generally highly consistent across studies, which suggests that our estimated average effect sizes might be fairly accurate summaries of the individual study effects.

### Limitations and future directions

Despite providing novel evidence that dominant group members can have more rather than less extreme affective reactions to witnessing dominant-on-disadvantaged as compared to dominant-on-dominant ostracism, our studies are not without their limitations. In particular, all of our studies utilized the same manipulation of ostracism (i.e., Cyberball). While Cyberball was selected because it is well validated as an effective manipulation of direct and vicarious ostracism, it is unclear whether similar, or conversely, weaker or stronger effects might be obtained with other manipulations. Indeed, research with targets of ostracism suggests that not all ostracism manipulations produce the same effects [92]. Thus, it would be worthwhile for future studies to examine the effects that are evident with alternate ostracism manipulations.

Similarly, in each study we only had a single control condition involving observing inclusion. Although we consider inclusion to be the most appropriate comparison for identifying the effects of ostracism, and indeed inclusion is typically the control condition in ostracism research, the extent to which the effects we obtained are unique to observing ostracism is currently unclear. It is possible that similar effects might be found when members of a dominant group witness fellow dominant group members perpetrate other forms of mistreatment against a disadvantaged group member. Yet, in light of past research documenting that dominant group members often do not react to blatant racism [36,37], our results may suggest that observing ostracism, perhaps because it is such a ubiquitous and relatable experience, might set the stage for enhanced sensitivity to prejudice and injustice. However, this interpretation is tentative and should be evaluated in future research.

Further, throughout all of our studies dominant and disadvantaged group status was defined only by ethnic background and the specific ethnic background of the disadvantaged target did not vary. Although this design decision allowed us to examine how members of a specific dominant ethnic group (i.e., White individuals) react to witnessing the ostracism of members of a particular historically disadvantaged ethnic group (e.g., Black individuals), the generalizability of our findings across other dominant-disadvantaged group relations remains an open question. Indeed, it is possible that dominant group members might be more upset by the ostracism of a Black person relative to members of other ethnic groups. Consistent with this possibility, recent surveys indicate that a majority of Americans view being Black as putting people at a disadvantage in American society, more so than other ethnic groups (e.g., Asian or Native Americans [93]). Dominant group members may also be more sensitive to the ostracism of a disadvantaged group member when disadvantage is defined by ethnic background as opposed to other group memberships (e.g., social class [45]). Relatedly, across studies the dominant versus disadvantaged status of the target overlapped with the ingroup versus outgroup status of the target. Thus, it is not clear that the effects we obtained result from the disadvantaged status or the outgroup status of the target. That some prior research using a minimal group paradigm has found more compensatory positive behavior directed toward excluded *ingroup* (vs. outgroup) members [39]–which is suggestive of greater negative reactivity to exclusion of ingroup members–perhaps speaks to the importance of disadvantage in driving our effects. However, this conclusion is speculative and awaits further testing. Consequently, to resolve these issues it would be beneficial for future studies to vary the ethnicity of the target as well as define advantage and disadvantage along different dimensions, particularly while holding the ingroup-outgroup status of the target constant.

We also acknowledge the real possibility, especially in view of the number of measures that were administered, that individuals’ responses to some of the items may have been affected by their previous exposure to other questions. Along these lines it is perhaps reassuring that although the affect measures sometimes appeared after the impression and empathy items (Studies 1 to 3) and sometimes before (Study 4), and a thought-listing task was sometimes presented right after the Cyberball game (Studies 2 to 4) and sometimes was not (Study 1), there was no evidence of significant heterogeneity in the effects on sadness or guilt across the studies. At the same time, however, it is also notable that the effects on anger were stronger in Study 4, the only study in which affect was measured first, than in the other studies. Because Study 4 differed from the others in a number of ways (including the specific items in the anger index) it is not possible to draw clear conclusions about the role of order of presentation from the present data. Indeed the possibilities for different types of order effects–both in the present research and more broadly in the literature on observed ostracism and observed discrimination–are extensive and complex. For example, perhaps stronger (or weaker) behavioral effects would have been obtained if the behavioral measures had been administered before the affect items. This issue, which has yet to undergo systematic study in the literature addressing reactions to ostracism, is an important one for future research to address.

Finally, all of our studies took place online. While the anonymity granted to participants by the online context likely helped alleviate social desirability concerns, it is possible that effects might be different if observers encountered the ostracism of an outgroup member perpetrated by their ingroup in person.

## Conclusion

Across four large experiments in which individuals perceived that they were engaging in an online interaction, we found that dominant group members reported greater feelings of guilt, sadness, and anger after observing their ingroup perpetrate ostracism against a member of a disadvantaged outgroup as opposed to an ingroup member. Moreover, we found no evidence that dominant group members experienced lower levels of empathy toward disadvantaged group as compared to dominant group targets of ostracism. Although past work has documented deficits in empathizing with outgroups and apathetic responses to overtly racist comments on the part of dominant group members, our results suggest that observing ostracism may be a sufficiently potent and relatable experience that when it occurs across group boundaries it awakens individuals’ sensitivity to injustice and discrimination.

## Supporting information

S1 File Contains additional methodological and analytic details. (DOCX)
