# Supplementary material for: Do dominant group members have different emotional responses to observing dominant-on-dominant versus dominant-on-disadvantaged ostracism? Some evidence for heightened reactivity to potentially discriminatory ingroup behavior
Source: PLoS One. 2020 Jun 25;15(6):e0234540. doi: 10.1371/journal.pone.0234540 (PMC7316269; doi:10.1371/journal.pone.0234540)
Supplement: S1 File — (DOCX) [file pone.0234540.s001.docx]

**Additional methodological and analytic details**

**Table of contents**

1. Results of Analysis of Variance Analyses (ANOVA) for Studies 1-3 in Mini Meta-Analysis

2. Additional Measures Collected from Participants

3. Results for Additional Measures: Target Compensation, Perceived Fairness, and Collective

Shame

4. Tables S1-S4: Mini Meta-Analysis of Additional Measures: Target Compensation, Perceived

Fairness, and Collective Shame

5. Details Regarding Coding of Participant Suspicion

6. Table S5: Participants Identified as Suspicious Across Studies 2 to 4

7. Tables S6-S9: Mini Meta-Analysis Excluding Participants Suspicious about Cyberball

8. Tables S10-S13: Mini Meta-Analysis Excluding Participants Suspicious about Study Purpose

9. Results of Observed Treatment × Target Type × Participant Sex ANOVAs for Studies 3 to 4

10. Assessment of Social Desirability

11. Tables S14-S17: Mini Meta-Analysis with Impression Management Covariate

**1. Results of analysis of variance analyses (ANOVA) for studies 1-3 in mini meta-analysis**

**Study 1**

**Affective reactions**

Analyses of participants’ affective reactions revealed a marginally significant Observed Treatment **×** Target Type interaction for sadness, *F*(1, 297) = 3.70, *p* = .055, $\text{η}_{\text{p}}^{\text{2}}$ = .012, 90% CI [.000, .041]^1^. Although the simple effect of observed treatment was significant for both White and Black targets, such that for both feelings of sadness were greater after observing severe ostracism relative to inclusion, this difference was larger when the target was a Black individual (*M*_severe ostracism_ = 3.93, *SD* = 2.51 vs. *M*_inclusion_ = 1.88, *SD* = 1.53), *F*(1, 297) = 32.36, *p* < .001, $\text{η}_{\text{p}}^{\text{2}}$ = .098, 90% CI [.051, .154], as opposed to an White individual (*M*_severe ostracism_ = 3.38, *SD* = 2.53 vs. *M*_inclusion_ = 2.31, *SD* = 2.12), *F*(1, 297) = 9.05, *p* = .003, $\text{η}_{\text{p}}^{\text{2}}$ = .030, 90% CI [.006, .068]. For all affective reactions there was a main effect of observed treatment, such that after observing severe ostracism relative to inclusion, participants felt more guilt, anger, sadness and less positive affect (*F*s ranged from 35.14 to 60.54, all *p*s < .001).

**Feelings of empathy toward target**

For participants’ feelings of empathy toward the target there was a significant main effect of observed treatment, such that participants empathized more with severely ostracized (*M* = 4.77, *SD* = 1.95) relative to included targets (*M* = 3.37, *SD* = 1.59), *F*(1, 297) = 46.55, *p* < .001, $\text{η}_{\text{p}}^{\text{2}}$ = .136, 90% CI [.080, .196].

**Impressions of target**

A marginally significant main effect of target type was evident on participants’ overall impressions of the target, with participants reporting more positive impressions of White (*M* = 3.92, *SD* = 1.01) relative to Black targets (*M* = 3.71, *SD* = 1.05), *F*(1, 297) = 3.04, *p* = .082, $\text{η}_{\text{p}}^{\text{2}}$ = .010, 90% CI [.000 .037].

**Study 2**

**Affective reactions**

Analyses of participants’ affective reactions yielded a marginally significant Observed Treatment **×** Target Type interaction for sadness, *F*(2, 917) = 2.56, *p* = .078, $\text{η}_{\text{p}}^{\text{2}}$ = .006, 90% CI [.000, .015]. Follow-up analyses indicated that the simple effect of target type, whereby participants reported greater feelings of sadness when the target was a Black relative to an White individual, was significant in the moderate ostracism condition (*M*_White/European_ = 2.28, *SD* = 2.08 vs. *M*_Black_ = 2.84, *SD* = 2.50), *F*(1, 917) = 4.48, *p* = .035, $\text{η}_{\text{p}}^{\text{2}}$ = .005, 90% CI [.000, .015] ) and was marginally significant in the severe ostracism condition (*M*_White/European_ = 3.08, *SD* = 2.63 vs. *M*_Black_ = 3.51, *SD* = 2.58), *F*(1, 917) = 2.94, *p* = .087, $\text{η}_{\text{p}}^{\text{2}}$ = .003, 90% CI [.000, .012], but was not evident in the inclusion condition (*M*_White/European_ = 1.98, *SD* = 1.86 vs. *M*_Black_ = 1.75, *SD* = 1.61), *F*(1, 917) = 0.75, *p* = .388, $\text{η}_{\text{p}}^{\text{2}}$ = .001, 90% CI [.000, .007]. For all affective reactions there was a significant main effect of observed treatment (*F*s ranged from 15.77 to 43.42, all *p*s < .001). Post-hoc comparisons revealed that for all affective reactions the moderate ostracism and severe ostracism conditions significantly differed from the inclusion condition, and that the two ostracism conditions also significantly differed from one another. For participants’ feelings of guilt, anger, and sadness, the mean levels of each affective reaction increased from the inclusion to the severe ostracism condition, whereas for positive affect the mean levels decreased from the inclusion to the severe ostracism condition. The main effect of target type was significant for feelings of anger, and marginally significant for guilt and sadness, such that participants reported greater feelings of anger, guilt, and sadness when the target was a Black relative to a White individual, *F*(1, 917) = 11.72, *p* = .001, $\text{η}_{\text{p}}^{\text{2}}$ = .013, 90% CI [.003, .027], *F*(1, 917) = 3.24, *p* = .072, $\text{η}_{\text{p}}^{\text{2}}$ = .004, 90% CI [.000, .013], and *F*(1, 917) = 2.83, *p* = .093, $\text{η}_{\text{p}}^{\text{2}}$ = .003, 90% CI [.000, .012] for anger, guilt, and sadness respectively (*F* < 1 for positive affect).

**Feelings of empathy toward target**

For participants’ feelings of empathy toward the target a significant Observed Treatment **×** Target Type interaction emerged, *F*(2, 918) = 5.52, *p* = .004, $\text{η}_{\text{p}}^{\text{2}}$ = .012, 90% CI [.002, .025]. Simple effects tests showed that the effect of target type, whereby participants empathized more with Black relative to White targets was significant in the moderate ostracism condition (*M*_Black_ = 5.15, *SD* = 1.63 vs. *M*_White/European_ = 4.53, *SD* = 1.72), *F*(1, 918) = 9.89, *p* = .002, $\text{η}_{\text{p}}^{\text{2}}$ = .011, 90% CI [.002, .024], but was not significant in the inclusion condition (*M*_White/European_ = 4.30, *SD* = 1.83 vs. *M*_Black_ = 3.25, *SD* = 1.77), *F* < 1 or the severe ostracism condition (*M*_White/European_ = 5.65, *SD* = 1.54 vs. *M*_Black_ = 5.39, *SD* = 1.74), *F*(1, 918) = 1.80, *p* = .180, $\text{η}_{\text{p}}^{\text{2}}$ = .002, 90% CI [.000, .010]. A significant main effect of observed treatment was also evident, *F*(2, 918) = 41.68, *p* < .001, $\text{η}_{\text{p}}^{\text{2}}$ = .083, 90% CI [.056, .112], which was qualified by the aforementioned interaction.

**Impressions of target**

There was a marginally significant main effect of target type, with participants reporting more positive impressions of Black (*M* = 4.54, *SD* = 1.14) compared to White targets (*M* = 4.40, *SD* = 1.19), *F*(1, 918) = 3.71, *p* = .054, $\text{η}_{\text{p}}^{\text{2}}$ = .004, 90% CI [.000, .014].

**Study 3**

**Affective reactions**

Analyses of participants’ affective reactions revealed a marginally significant Observed Treatment **×** Target Type interaction for feelings of guilt, *F*(2, 1952) = 3.00, *p* = .050, $\text{η}_{\text{p}}^{\text{2}}$ = .003, 90% CI [.000, .008]. Follow-up analyses indicated that the simple effect of target type was significant only in the severe ostracism condition, such that after observing severe ostracism participants experienced greater feelings of guilt when the target was a Black (*M* = 1.39, *SD* = 0.75) as opposed to an White individual (*M* = 1.23, *SD* = 0.57), *F*(1, 1952) = 10.62, *p* = .001, $\text{η}_{\text{p}}^{\text{2}}$ = .005, 90% CI [.001, .012]; for the inclusion condition (*M*_White/European_ = 1.22, *SD* = 0.64 vs. *M*_Black_ = 1.21, *SD* = 0.57) and moderate ostracism condition (*M*_White/European_ = 1.22, *SD* = 0.53 vs. *M*_Black_ = 1.26, *SD* = 0.63), both *F*s < 1. Significant main effects of observed treatment and target type, both qualified by the above interaction, were also evident on guilt, *F*(2, 1952) = 4.12, *p* = .016, $\text{η}_{\text{p}}^{\text{2}}$ = .004, 90% CI [.000, .010] and *F*(1, 1952) = 5.57, *p* = .018, $\text{η}_{\text{p}}^{\text{2}}$ = .003, 90% CI [.000, .008], respectively. For anger and sadness a significant main effect of observed treatment was evident, *F*(2, 1952) = 14.48, *p* < .001, $\text{η}_{\text{p}}^{\text{2}}$ = .015, 90% CI [.007, .024] and *F*(2, 1952) = 30.07, *p* < .001, $\text{η}_{\text{p}}^{\text{2}}$ = .030, 90% CI [.018, .043], respectively. For both anger and sadness post-hoc comparisons indicated that all conditions significantly differed from one another (all *p*s < .01) with participants increasingly feeling more anger and sadness the more negatively the target was treated.

**Feelings of empathy toward target**

A significant main effect of observed treatment was evident on participants’ feelings of empathy toward the target, *F*(2, 1952) = 97.61, *p* < .001, $\text{η}_{\text{p}}^{\text{2}}$ = .091, 90% CI [.071, .111]. Post-hoc tests comparing the observed treatment conditions indicated that all of the observed treatment conditions significantly differed from one another (all *p*s < .001), such that participants increasingly empathized with targets the more negatively they were treated. A marginally significant main effect of target type was also evident, with participants empathizing more with Black (*M* = 4.65, *SD* = 1.71) relative to White targets (*M* = 5.20, *SD* = 1.73), *F*(1, 1952) = 3.51, *p* = .061, $\text{η}_{\text{p}}^{\text{2}}$ = .002, 90% CI [.000, .006].

**Impressions of target*.***

Analyses of participants’ overall impressions of the target revealed a significant Observed Treatment **×** Target Type interaction, *F*(2, 1952) = 3.54, *p* = .029, $\text{η}_{\text{p}}^{\text{2}}$ = .004, 90% CI [.000, .009]. Simple effects tests indicated that the effect of target type was significant only in the inclusion condition, such that after observing inclusion participants reported more positive impressions of Black (*M* = 4.47, *SD* = 1.11) as opposed to White targets (*M* = 4.22, *SD* = 1.21), *F*(1, 1952) = 7.61, *p* = .006, $\text{η}_{\text{p}}^{\text{2}}$ = .004, 90% CI [.001, .010]; for the moderate ostracism condition (*M*_White/European_ = 4.28, *SD* = 1.21 vs. *M*_Black_ = 4.38, *SD* = 1.11), *F*(1, 1952) = 1.20, *p* = .273, $\text{η}_{\text{p}}^{\text{2}}$ = .001, 90%. CI [.000, .004]; for the severe ostracism condition (*M*_White/European_ = 4.42, *SD* = 1.24 vs. *M*_Black_ = 4.33, *SD* = 1.22), *F*(1, 1952) = 1.00, *p* = .318, $\text{η}_{\text{p}}^{\text{2}}$ = .001, 90% CI [.000, .004].

**2. Additional measures collected from participants**

Across studies participants completed several other measures in addition to those

described in the main text. These additional measures are outlined separately for each study

below.

**Study 1**

Embedded among the impression dimensions that immediately followed Cyberball was one item measuring the perceived fairness of each player’s behavior. This item asked participants to indicate how fair they thought the behavior of each player was (1 = *unfair*, 7 = *fair*). We focused on participants’ ratings of the fairness of the actions of the two perpetrators.

The remaining additional measures followed our assessment of participants’ affect and were completed in the order in which they are presented below.

Participants completed the Revised Philosophies of Human Nature scale (R-PHN; [2,3]), which consists of the Cynicism (“Most people will tell a lie if they could gain by it”) and Belief that People are Conventional Good (“The typical person is sincerely concerned about the problems of others”) subscales. Ratings were made on a 6-point scale (-3 = *strongly disagree*, +3 = *agree strongly*).

Participants also completed the Trust in People scale [3,4]. The Trust in People scale consisted of the following three items: “Generally speaking, would you say that most people can be trusted or that you can’t be too careful in dealing with people?”, “Would you say that most of the time, people try to be helpful, or that they are mostly just looking out for themselves?”, and “Do you think that most people would try to take advantage of you if they got the chance or would they try to be fair?”. These items were forced-choice and asked participants to select either a negative (coded as 1) or positive (coded as 2) option (e.g., “You cannot be too careful” vs. “Most people can be trusted”).

We also assessed current feelings of attachment security and insecurity with the State Adult Attachment Measure (SAAM [5]). The SAAM contains three subscales: security (“I feel like I have someone to rely on”), anxiety (“I really need to feel loved right now”), and avoidance (“If someone tried to get close to me, I would try to keep my distance”). Ratings were made on a 7-point scale (1 = *disagree strongly*, 7 = *agree strongly*).

We measured the degree to which participants sought to compensate the target for how they were treated during the witnessed interaction by having them configure a task that the ostensible other participants from Cyberball were asked to complete. They were informed that in this task, the other participants would act as business-owners and engage in an exchange of resources with one another with the goal of maximizing their own stockpile of resources. They were further notified that participants selected as observers for Cyberball were tasked with designating the starting number of resources for each player out of a total of 15. Participants were also told that their designation of resources would be anonymous to the other participants. The total number of resources provided to the player who was the target in Cyberball was used as our measure of target compensation.

The final additional measure consisted of four items, embedded among demographics at the end of the survey, which evaluated feelings of collective shame. These items were adapted from Branscombe et al. [6] and Piff et al. [7]. The four items were: “I feel shame for my group’s harmful past actions toward other groups”, “I feel shame about the negative things my ancestors did to other groups”, “I feel shame for some of the things my group did to other groups in the past”, and “I can easily feel shame for the bad outcomes brought about by members of my group”. Ratings were made on a 7-point scale (1 = *strongly disagree*, 7 = *strongly agree*).

**Study 2**

A number of the supplementary measures in Study 2 overlapped with those from Study 1, including perceived fairness of perpetrators’ behavior, the Cynicism subscale of the R-PHN, the SAAM, as well as the measure of target compensation and collective shame. These items were completed by participants in the same order as Study 1. However, in addition, Study 2 contained a behavioral measure that was unique to that study and asked participants to select the role (observer or player) they would prefer for an ostensible second game of Cyberball. This item appeared after the target compensation measure.

**Study 3 and Study 4**

The additional measure in Studies 3 and 4 was the perceived fairness of the perpetrators’ actions. However, unlike in Studies 1 or 2, the measure of perceived fairness was expanded by adding two additional items which asked participants to indicate how unbiased (1 = *biased*, 7 = *unbiased*) and open-minded (1 = *closed-minded*, 7 = *open-minded*) they perceived each player to have acted.

**3. Results for additional measures: Target compensation, perceived fairness, and collective shame**

Below we report meta-analytic results for the additional measures, namely target compensation, perceived fairness of the perpetrators’ actions, and collective shame, that are most theoretically relevant to the focus of the research presented in the main text (see Tables S1-S4). Some of these results, although weak, are broadly consistent with those reported in the main text. For example, the effect of target type on collective shame tended to differ across the inclusion and severe ostracism conditions, with a descriptively positive effect of the target being Black rather than White in the severe ostracism condition and a descriptively negative effect of the target being Black rather than White in the inclusion condition. That said, the effects involving target ethnicity were weak and generally did not reach conventional levels of statistical significance.

The absence of the expected pattern for target compensation might be explained by participants thinking that the divvying of points for an experimental task was not particularly consequential or likely to have much of a tangible impact on the disadvantaged group target. Alternatively or in addition, it is possible that the method of compensation was sufficiently easy that it was not as sensitive a measure as decisions about sending messages and what to write: These decisions and actions were comparatively more effortful – such that they depended more on participants’ current affect and motivations – and may have been seen by participants as more impactful as well.

The lack of specificity of our fairness items might account for the weak effects here. Specifically, because we did not want to explicitly cue participants to our focus on race the items assessing fairness measured perceptions of fairness more generally rather than perceptions of racism or discrimination per se. This choice may have reduced the sensitivity of our fairness measure and consequently may have undercut any potential moderation by target type of the strong main effect of observed treatment.

Finally, the lack of significant effects for collective shame might simply be a result of these items being completed at the very end of their respective studies. Like the wording of the fairness items this decision was made in an effort to reduce the degree to which participants developed suspicions about the purpose of the study. However, it is possible that the impact of observing ostracism may have dissipated somewhat by the time participants reported their feelings of collective shame. Indeed, the effect of witnessing the target’s mistreatment may have been partially assuaged by the opportunity for participants to state their affect, impressions, and so forth. As a result, the placement of these items within the study may have reduced their sensitivity.

| Table S1 | | | | | | | | | |
| --- | --- | --- | --- | --- | --- | --- | --- | --- | --- |
| *Overall Effect of Target Type for Additional Measures: Target Compensation, Perceived Fairness, and Collective Shame in Each Observed Treatment Condition* | | | | | | | | | |
| Measure/condition | Inclusion  (Studies 1 to 4) | | | Moderate ostracism  (Studies 2 and 3 only) | | | Severe ostracism  (Studies 1 to 4) | | |
|  | *d* 95% CI | *Q* | *I*^2^ | *d* 95% CI | *Q* | *I*^2^ | *d* 95% CI | *Q* | *I*^2^ |
| Additional measures | | | | | | | | | |
| Target compensation | 0.06 [-0.13, 0.24] | 1.69 | 40.90 | 0.11^b^ [-0.12, 0.33] | -- | -- | -0.09 [-0.27, 0.10] | 1.94 | 48.46 |
|  |  |  |  |  |  |  |  |  |  |
|  |  |  |  |  |  |  |  |  |  |
|  |  |  |  |  |  |  |  |  |  |
|  |  |  |  |  |  |  |  |  |  |
|  |  | | |  | | |  | | |
| Perceived fairness of the perpetrators’ actions | **0.11* [0.01, 0.20]** | 0.59 | 0.00 | 0.09 [-0.03, 0.22] | 5.16* | 80.62 | -0.04 [-0.13, 0.06] | 11.69** | 74.34 |
|  |  |  |  |  |  |  |  |  |  |
|  |  | | |  | | |  | | |
|  |  |  |  |  |  |  |  |  |  |
|  |  |  |  |  |  |  |  |  |  |
|  |  | | |  | | |  | | |
| Collective shame | -0.10 [-0.28, 0.09] | 0.22 | 0.00 | 0.11^b^ [-0.12, 0.34] | -- | -- | 0.14 [-0.04, 0.32] | 3.91* | 74.42 |
| *Note.* Positive Cohen’s *d*s indicate greater reactivity toward Black targets (i.e., higher mean scores on a dependent measure when target was a Black individual), whereas negative Cohen’s *d*s indicate greater reactivity to White/European targets. Cohen’s *d* was computed using a fixed-effects model*.* CI = confidence interval; *Q* = Cochran’s *Q* and *I*^2^ =  *I*^2^ index used to test for and quantify the degree of heterogeneity in effect sizes, respectively. ^b^ = Cohen’s *d* based on a single study. † *p* < .10 * *p* < .05 ** *p* < .01 *** *p* < .001 | | | | | | | | | |

| Table S2 | | | | | | | |  | |  | |
| --- | --- | --- | --- | --- | --- | --- | --- | --- | --- | --- | --- |
| *Overall Effect of Target Type and Tests of Moderation for Additional Measures: Target Compensation, Perceived Fairness, and Collective Shame* | | | | | | | | | | | |
|  |  | | |  | | Contrast | | | | | |
| Measure | Effect across conditions | | | Omnibus moderation | | moderate vs. inclusion | | severe vs. inclusion | | severe vs. moderate | |
|  | *d* 95% CI | *Q* | *I*^2^ | *QM*(*df*) | | *B* | *SE* | *B* | *SE* | *B* | *SE* |
| Additional measures | | | | | | | | | | | |
| Target compensation | 0.01 [-0.10, 0.13] | 5.65 | 29.14 | 2.01(2) | | 0.05 | 0.15 | -0.14 | 0.13 | -0.19 | 0.15 |
|  |  |  |  |  |  |  |  |  |  |  |  |
|  |  |  |  |  |  |  |  |  |  |  |  |
|  |  |  |  |  | |  |  |  |  |  |  |
|  |  |  |  |  |  |  |  |  |  |  |  |
|  |  |  |  |  |  |  |  |  |  |  |  |
| Perceived fairness of the perpetrators’ actions | 0.05 [-0.01, 0.11] | 22.60** | 60.17 | **5.15(2)**† | | -0.01 | 0.08 | **-0.14*** | 0.07 | **-0.13†** | 0.08 |
|  |  |  |  |  |  |  |  |  |  |  |  |
|  |  |  |  |  |  |  |  |  |  |  |  |
|  |  |  |  |  | |  |  |  |  |  |  |
|  |  |  |  |  |  |  |  |  |  |  |  |
|  |  |  |  |  |  |  |  |  |  |  |  |
| Collective shame | 0.05 [-0.07, 0.16] | 7.78† | 48.58 | 3.65(2) | | 0.21 | 0.15 | **0.24†** | 0.13 | 0.03 | 0.15 |
| *Note.* Positive Cohen’s *d*s indicate greater reactivity toward Black targets (i.e., higher mean scores on a dependent measure when target was a Black individual), whereas negative Cohen’s *d*s indicate greater reactivity to White/European targets. Cohen’s *d* was computed using a fixed-effects model. CI = confidence interval; *Q* = Cochran’s *Q* and *I*^2^ =  *I*^2^ index used to test for and quantify the degree of heterogeneity in effect sizes, respectively; *QM* = omnibus test of moderator model coefficients; *df* = degrees of freedom; *SE* = standard error. † *p* < .10 * *p* < .05 ** *p* < .01 *** *p* < .001 | | | | | | | | | | | |

| Table S3 | | | | | | | |
| --- | --- | --- | --- | --- | --- | --- | --- |
| *Overall Effect of Observed Treatment for Additional Measures: Target Compensation, Perceived Fairness, and Collective Shame for each Target Type* | | | | | | | |
| Measure/condition | White/European target | | |  | Black target | | |
|  | *d* 95% CI | *Q* | *I*^2^ |  | *d* 95% CI | *Q* | *I*^2^ |
| Affective reactions | | | | | | | |
| Target compensation | **0.45*** [0.28, 0.62]** | 2.23 | 55.16 |  | **0.40*** [0.23, 0.57]** | 1.64 | 39.20 |
|  |  |  |  |  |  |  |  |
|  |  |  |  |  |  |  |  |
|  |  |  |  |  |  |  |  |
|  |  |  |  |  |  |  |  |
|  |  | | |  |  | | |
| Perceived fairness of  the perpetrators’ actions | **-1.60*** [-1.70, -1.50]** | 13.96** | 78.50 |  | **-1.60*** [-1.70, -1.50]** | 49.32*** | 93.92 |
|  |  |  |  |  |  |  |  |
|  |  | | |  |  | | |
|  |  |  |  |  |  |  |  |
|  |  |  |  |  |  |  |  |
|  |  | | |  |  | | |
| Collective shame | -0.14 [-0.30, 0.03] | 3.47† | 71.17 |  | 0.11 [-0.05, 0.28] | 0.33 | 0.00 |
| *Note.* Positive Cohen’s *d*s indicate greater reactivity to ostracism (i.e., higher mean scores on a dependent measure when the target was ostracized), whereas negative Cohen’s *d*s indicate greater reactivity to inclusion. Cohen’s *d* was computed using a fixed-effects model. The overall effect of observed treatment collapses across the moderate and severe ostracism conditions*.* CI = confidence interval; *Q* = Cochran’s *Q* and *I*^2^ =  *I*^2^ index used to test for and quantify the degree of heterogeneity in effect sizes, respectively. † *p* < .10 * *p* < .05 ** *p* < .01 *** *p* < .001 | | | | | | | |

| Table S4 | | | | | | | |
| --- | --- | --- | --- | --- | --- | --- | --- |
| *Overall Effect of Observed Treatment and Tests of Moderation for Additional Measures: Target Compensation, Perceived Fairness, and Collective Shame* | | | | | | | |
|  |  | | |  | | Contrast | |
| Measure | Effect across target type | | | Omnibus moderation | | Black vs. White/European target | |
|  | *d* 95% CI | *Q* | *I*^2^ | *QM*(*df*) | | *B* | *SE* |
| Affective reactions | | | | | | | |
| Target compensation | **0.42*** [0.30, 0.54]** | 4.04 | 25.78 | 0.17(1) | | -0.05 | 0.12 |
|  |  |  |  |  |  |  |  |
|  |  |  |  |  |  |  |  |
|  |  |  |  |  | |  |  |
|  |  |  |  |  |  |  |  |
|  |  |  |  |  |  |  |  |
| Perceived fairness of  the perpetrators’ actions | **-1.60*** [-1.67, -1.53]** | 63.28*** | 88.94 | 0.003(1) | | 0.004 | 0.07 |
|  |  |  |  |  |  |  |  |
|  |  |  |  |  |  |  |  |
|  |  |  |  |  | |  |  |
|  |  |  |  |  |  |  |  |
|  |  |  |  |  |  |  |  |
| Collective shame | -0.01 [-0.13, 0.11] | 8.07* | 62.83 | **4.28(1)*** | | **0.25*** | 0.12 |
| *Note.* Positive Cohen’s *d*s indicate greater reactivity to ostracism (i.e., higher mean scores on a dependent measure when the target was ostracized), whereas negative Cohen’s *d*s indicate greater reactivity to inclusion. Cohen’s *d* was computed using a fixed-effects model. The overall effect of observed treatment collapses across the moderate and severe ostracism conditions. CI = confidence interval; *Q* = Cochran’s *Q* and *I*^2^ =  *I*^2^ index used to test for and quantify the degree of heterogeneity in effect sizes, respectively; *QM* = omnibus test of moderator model coefficients; *df* = degrees of freedom; *SE* = standard error. * *p* < .05 ** *p* < .01 *** *p* < .001 | | | | | | | |

**5. Details regarding coding for participant suspicion**

In Studies 2 through 4 suspicion was coded by the authors from an open-ended thought-listing that immediately followed the game of Cyberball. The thought-listing asked participants to list the thoughts and feelings that they had while observing the game between the other ostensible participants. The authors were blind to participants’ condition assignments while completing the coding. In addition to coding whether participants indicated that they thought the game of Cyberball or its components (e.g., photos of the other players) were likely contrived, we expanded the definition of suspicion from our pre-registrations to also include whether participants believed the likely purpose of the study was to evaluate racism, prejudice, or discrimination. These judgments were coded dichotomously (0 = no; 1 = yes). The average level of agreement across studies was high for both dimensions. Cohen’s Kappa averaged $\text{κ}$ = .74 for suspicion about the authenticity of Cyberball (ranged from $\text{κ}$ = .71 to .82) and $\text{κ}$ = .75 for suspicion about the purpose of the study (ranged from $\text{κ}$ = .70 to .78). For either dimension participants were identified as suspicious only if authors’ ratings agreed. It should be noted that we did not code suspicion in Study 1 because that study did not contain the thought-listing.

| Table S5 | | | | | | | | | |
| --- | --- | --- | --- | --- | --- | --- | --- | --- | --- |
| *Participants Identified as Suspicious Across Studies 2 to 4* | | | | | | | | | |
| Study | Suspicious about Cyberball | | | |  | Suspicious about study purpose | | | |
|  | *n* | *N* | % | *N* after exclusions |  | *n* | *N* | % | *N* after exclusions |
| 2 | 32 | 924 | 3.5 | 892 |  | 11 | 924 | 1.2 | 913 |
| 3 | 87 | 1958 | 4.4 | 1871 |  | 21 | 1958 | 1.1 | 1937 |
| 4 | 52 | 1230 | 4.2 | 1178 |  | 19 | 1230 | 1.5 | 1211 |
| *Note*. Suspicious about Cyberball denotes participants who were rated by both coders as indicating in an open-ended thought-listing that followed Cyberball that they thought the game of Cyberball or its components were likely contrived. Suspicious about study purpose denotes participants who were rated by both coders as indicating that they believed the likely purpose of the study or game of Cyberball was to evaluate racism, prejudice, or discrimination. | | | | | | | | | |

| Table S6 | | | | | | | | | |
| --- | --- | --- | --- | --- | --- | --- | --- | --- | --- |
| *Overall Effect of Target Type for Affective Reactions, Empathy Toward Target, and Impressions of Target in Each Observed Treatment Condition with Participants Suspicious about Cyberball Excluded* | | | | | | | | | |
| Measure/condition | Inclusion  (Studies 1 to 4) | | | Moderate ostracism  (Studies 2 and 3 only) | | | Severe ostracism  (Studies 1 to 4) | | |
|  | *d* 95% CI | *Q* | *I*^2^ | *d* 95% CI | *Q* | *I*^2^ | *d* 95% CI | *Q* | *I*^2^ |
| Affective reactions | | | | | | | | | |
| Guilt | -0.06 [-0.16, 0.03] | 0.79 | 0.00 | **0.12† [-0.01, 0.25]** | 0.62 | 0.00 | **0.18*** [0.09, 0.28]** | 1.66 | 0.00 |
|  |  |  |  |  |  |  |  |  |  |
|  |  |  |  |  |  |  |  |  |  |
| Anger | -0.07 [-0.17, 0.02] | 6.73† | 55.40 | 0.09 [-0.03, 0.22] | 10.07** | 90.07 | **0.15** [0.05, 0.25]** | 9.48* | 68.35 |
|  |  |  |  |  |  |  |  |  |  |
|  |  | | |  | | |  | | |
| Sadness | -0.07 [-0.16, 0.03] | 2.18 | 0.00 | 0.09 [-0.04, 0.22] | 3.36† | 70.23 | **0.13** [0.04, 0.23]** | 0.44 | 0.00 |
|  |  |  |  |  |  |  |  |  |  |
|  |  | | |  | | |  | | |
| Fear | -0.05 [-0.17, 0.06] | 2.73† | 63.36 | -0.09^b^ [-0.24, 0.07] | -- | -- | 0.08 [-0.03, 0.20] | 0.08 | 0.00 |
|  |  |  |  |  |  |  |  |  |  |
|  |  | | |  | | |  | | |
| Positive affect | -0.003 [-0.10, 0.09] | 1.61 | 0.00 | -0.04 [-0.17, 0.09] | 0.11 | 0.00 | -0.01 [-0.11, 0.09] | 1.25 | 0.00 |
|  |  |  |  |  |  |  |  |  |  |
| Feelings of empathy | | | | | | | | | |
|  |  |  |  |  |  |  |  |  |  |
| Empathy toward target | **0.09† [-0.00, 0.19]** | 4.12 | 27.18 | **0.15* [0.02, 0.28]** | 6.26* | 84.04 | 0.04 [-0.05, 0.14] | 4.10 | 26.89 |
|  |  |  |  |  |  |  |  |  |  |
| Positivity of impressions of target | | | | | | | | | |
|  |  |  |  |  |  |  |  |  |  |
| Impressions of target | 0.02 [-0.08, 0.12] | 12.45** | 75.91 | **0.17** [0.04, 0.30]** | 2.17 | 53.99 | **-0.12* [-0.21, -0.02]** | 4.93 | 39.17 |
| *Note.* Positive Cohen’s *d*s indicate greater reactivity toward Black targets (i.e., higher mean scores on a dependent measure when target was a Black individual), whereas negative Cohen’s *d*s indicate greater reactivity to White/European targets. Cohen’s *d* was computed using a fixed-effects model*.* CI = confidence interval; *Q* = Cochran’s *Q* and *I*^2^ =  *I*^2^ index used to test for and quantify the degree of heterogeneity in effect sizes, respectively. ^b^ = Cohen’s *d* based on a single study. † *p* < .10 * *p* < .05 ** *p* < .01 *** *p* < .001 | | | | | | | | | |

| Table S7 | | | | | | | |  | |  | | |
| --- | --- | --- | --- | --- | --- | --- | --- | --- | --- | --- | --- | --- |
| *Overall Effect of Target Type and Tests of Moderation for Affective Reactions, Empathy Toward Target, and Impressions of Target with Participants Suspicious about Cyberball Excluded* | | | | | | | | | | | | |
|  |  | | |  | | Contrast | | | | | | |
| Measure | Effect across conditions | | | Omnibus moderation | | moderate vs. inclusion | | severe vs. inclusion | | severe vs. moderate | | |
|  | *d* 95% CI | *Q* | *I*^2^ | *QM*(*df*) | | *B* | *SE* | *B* | *SE* | *B* | *SE* | |
| Affective reactions | | | | | | | | | | | | |
| Guilt | **0.07* [0.01, 0.13]** | 16.42† | 45.18 | **13.36(2)**** | | **0.19*** | 0.08 | **0.25***** | 0.07 | 0.06 | 0.08 | |
|  |  |  |  |  |  |  |  |  |  |  |  | |
|  |  |  |  |  |  |  |  |  |  |  |  | |
| Anger | 0.05 [-0.01, 0.11] | 37.10*** | 75.74 | **10.83(2)**** | | **0.17*** | 0.08 | **0.22**** | 0.07 | 0.06 | 0.08 | |
|  |  |  |  |  |  |  |  |  |  |  |  | |
|  |  |  |  |  |  |  |  |  |  |  |  | |
| Sadness | 0.04 [-0.02, 0.10] | 14.71† | 38.82 | **8.72(2)*** | | **0.15†** | 0.08 | **0.20**** | 0.07 | 0.04 | 0.08 | |
|  |  |  |  |  |  |  |  |  |  |  |  | |
|  |  |  |  |  |  |  |  |  |  |  |  | |
| Fear | **-**0.01 [-0.08, 0.06] | 6.88 | 41.85 | 4.07(2) | | -0.03 | 0.10 | **0.14†** | 0.08 | **0.17†** | 0.10 | |
|  |  |  |  |  |  |  |  |  |  |  |  | |
|  |  |  |  |  |  |  |  |  |  |  |  | |
| Positive affect | **-**0.01 [-0.07, 0.05] | 3.16 | 0.00 | 0.19(2) | | -0.04 | 0.08 | -0.01 | 0.07 | 0.03 | 0.08 | |
|  |  |  |  |  |  |  |  |  |  |  |  |  |
| Feelings of empathy | | | | | | | | | | | | |
|  |  |  |  |  |  |  |  |  |  |  |  | |
| Empathy toward target | **0.09** [0.03, 0.15]** | 16.22† | 44.51 | 1.73(2) | | 0.06 | 0.08 | -0.05 | 0.07 | -0.11 | 0.08 | |
|  |  |  |  |  |  |  |  |  |  |  |  | |
| Positivity of impressions of target | | | | | | | | | | | | |
|  |  |  |  |  |  |  |  |  |  |  |  | |
| Impressions of target | 0.001 [-0.06, 0.06] | 32.11*** | 71.98 | **12.56(2)**** | | **0.15†** | 0.08 | **-0.14†** | 0.07 | **-0.29***** | 0.08 | |
| *Note.* Positive Cohen’s *d*s indicate greater reactivity toward Black targets (i.e., higher mean scores on a dependent measure when target was a Black individual), whereas negative Cohen’s *d*s indicate greater reactivity to White/European targets. Cohen’s *d* was computed using a fixed-effects model. CI = confidence interval; *Q* = Cochran’s *Q* and *I*^2^ =  *I*^2^ index used to test for and quantify the degree of heterogeneity in effect sizes, respectively; *QM* = omnibus test of moderator model coefficients; *df* = degrees of freedom; *SE* = standard error. † *p* < .10 * *p* < .05 ** *p* < .01 *** *p* < .001 | | | | | | | | | | | | |

| Table S8 | | | | | | | |
| --- | --- | --- | --- | --- | --- | --- | --- |
| *Overall Effect of Observed Treatment for Affective Reactions, Empathy Toward Target, and Impressions of Target for each Target Type with Participants Suspicious about Cyberball Excluded* | | | | | | | |
| Measure/condition | White/European target | | |  | Black target | | |
|  | *d* 95% CI | *Q* | *I*^2^ |  | *d* 95% CI | *Q* | *I*^2^ |
| Affective reactions | | | | | | | |
| Guilt | 0.07 [-0.02, 0.16] | 20.01*** | 84.06 |  | **0.29*** [0.20, 0.38]** | 23.97*** | 87.48 |
|  |  |  |  |  |  |  |  |
|  |  |  |  |  |  |  |  |
| Anger | **0.31*** [0.22, 0.40]** | 15.02** | 80.03 |  | **0.51*** [0.42, 0.60]** | 29.83*** | 89.94 |
|  |  |  |  |  |  |  |  |
|  |  | | |  |  | | |
| Sadness | **0.25*** [0.16, 0.34]** | 6.73† | 55.40 |  | **0.43*** [0.34, 0.52]** | 18.47*** | 83.75 |
|  |  |  |  |  |  |  |  |
|  |  | | |  |  | | |
| Fear | -0.03 [-0.13, 0.08] | 1.62 | 38.23 |  | 0.04 [-0.06, 0.15] | 2.39 | 58.17 |
|  |  |  |  |  |  |  |  |
|  |  | | |  |  | | |
| Positive affect | **-0.16* [-0.24, -0.07]** | 27.23*** | 88.98 |  | **-0.18*** [-0.27, -0.09]** | 14.14** | 78.78 |
|  |  |  |  |  |  |  |  |
| Feelings of empathy | | | | | | | |
|  |  |  |  |  |  |  |  |
| Empathy toward target | **0.52*** [0.43, 0.61]** | 19.48*** | 84.60 |  | **0.51*** [0.42, 0.60]** | 6.89† | 56.49 |
|  |  |  |  |  |  |  |  |
| Positivity of impressions of target | | | | | | | |
|  |  |  |  |  |  |  |  |
| Impressions of target | **0.12** [0.04, 0.21]** | 12.32** | 75.66 |  | 0.04 [-0.05, 0.13] | 9.96* | 69.88 |
| *Note.* Positive Cohen’s *d*s indicate greater reactivity to ostracism (i.e., higher mean scores on a dependent measure when the target was ostracized), whereas negative Cohen’s *d*s indicate greater reactivity to inclusion. Cohen’s *d* was computed using a fixed-effects model. The overall effect of observed treatment collapses across the moderate and severe ostracism conditions*.* CI = confidence interval; *Q* = Cochran’s *Q* and *I*^2^ =  *I*^2^ index used to test for and quantify the degree of heterogeneity in effect sizes, respectively. † *p* < .10 * *p* < .05 ** *p* < .01 *** *p* < .001 | | | | | | | |

| Table S9 | | | | | | | |
| --- | --- | --- | --- | --- | --- | --- | --- |
| *Overall Effect of Observed Treatment and Tests of Moderation for Affective Reactions, Empathy Toward Target, and Impressions of Target with Participants Suspicious about Cyberball Excluded* | | | | | | | |
|  |  | | |  | | Contrast | |
| Measure | Effect across target type | | | Omnibus moderation | | Black vs. White/European target | |
|  | *d* 95% CI | *Q* | *I*^2^ | *QM*(*df*) | | *B* | *SE* |
| Affective reactions | | | | | | | |
| Guilt | **0.18*** [0.11, 0.24]** | 53.89*** | 87.01 | **11.10(1)***** | | **0.21**** | 0.06 |
|  |  |  |  |  |  |  |  |
|  |  |  |  |  |  |  |  |
| Anger | **0.41*** [0.35, 0.47]** | 54.83*** | 87.23 | **9.98(1)**** | | **0.20**** | 0.06 |
|  |  |  |  |  |  |  |  |
|  |  |  |  |  |  |  |  |
| Sadness | **0.34*** [0.27, 0.40]** | 32.71*** | 78.60 | **7.52(1)**** | | **0.18**** | 0.06 |
|  |  |  |  |  |  |  |  |
|  |  |  |  |  |  |  |  |
| Fear | 0.01 [-0.07, 0.08] | 4.89 | 38.66 | 0.88(1) | | 0.07 | 0.08 |
|  |  |  |  |  |  |  |  |
|  |  |  |  |  |  |  |  |
| Positive affect | **-0.17*** [-0.23, -0.10]** | 41.49*** | 83.13 | 0.13(1) | | -0.02 | 0.06 |
|  |  |  |  |  |  |  |  |
| Feelings of empathy | | | | | | | |
|  |  |  |  |  | |  |  |
| Empathy toward target | **0.52*** [0.45, 0.58]** | 26.41*** | 73.50 | 0.04(1) | | -0.01 | 0.07 |
|  |  |  |  |  |  |  |  |
| Positivity of impressions of target | | | | | | | |
|  |  |  |  |  | |  |  |
| Impressions of target | **0.08* [0.02, 0.14]** | 24.04** | 70.88 | 1.75(1) | | -0.08 | 0.06 |
| *Note.* Positive Cohen’s *d*s indicate greater reactivity to ostracism (i.e., higher mean scores on a dependent measure when the target was ostracized), whereas negative Cohen’s *d*s indicate greater reactivity to inclusion. Cohen’s *d* was computed using a fixed-effects model. The overall effect of observed treatment collapses across the moderate and severe ostracism conditions. CI = confidence interval; *Q* = Cochran’s *Q* and *I*^2^ =  *I*^2^ index used to test for and quantify the degree of heterogeneity in effect sizes, respectively; *QM* = omnibus test of moderator model coefficients; *df* = degrees of freedom; *SE* = standard error. * *p* < .05 ** *p* < .01 *** *p* < .001 | | | | | | | |

| Table S10 | | | | | | | | | |
| --- | --- | --- | --- | --- | --- | --- | --- | --- | --- |
| *Overall Effect of Target Type for Affective Reactions, Empathy Toward Target, and Impressions of Target in Each Observed Treatment Condition with Participants Suspicious about the Purpose of the Studies Excluded* | | | | | | | | | |
| Measure/condition | Inclusion  (Studies 1 to 4) | | | Moderate ostracism  (Studies 2 and 3 only) | | | Severe ostracism  (Studies 1 to 4) | | |
|  | *d* 95% CI | *Q* | *I*^2^ | *d* 95% CI | *Q* | *I*^2^ | *d* 95% CI | *Q* | *I*^2^ |
| Affective reactions | | | | | | | | | |
| Guilt | -0.06 [-0.15, 0.04] | 1.00 | 0.00 | **0.11† [-0.02, 0.24]** | 0.80 | 0.00 | **0.19*** [0.10, 0.29]** | 1.85 | 0.00 |
|  |  |  |  |  |  |  |  |  |  |
|  |  |  |  |  |  |  |  |  |  |
| Anger | -0.06 [-0.16, 0.03] | 7.01† | 57.23 | 0.10 [-0.03, 0.22] | 9.28** | 89.23 | **0.16** [0.06, 0.25]** | 7.05† | 57.47 |
|  |  |  |  |  |  |  |  |  |  |
|  |  | | |  | | |  | | |
| Sadness | -0.06 [-0.16, 0.03] | 2.47 | 0.00 | 0.09 [-0.04, 0.21] | 2.70 | 62.90 | **0.14** [0.05, 0.24]** | 0.64 | 0.00 |
|  |  |  |  |  |  |  |  |  |  |
|  |  | | |  | | |  | | |
| Fear | -0.05 [-0.16, 0.06] | 2.64 | 62.13 | -0.09^b^ [-0.25, 0.06] | -- | -- | 0.08 [-0.03, 0.19] | 0.12 | 0.00 |
|  |  |  |  |  |  |  |  |  |  |
|  |  | | |  | | |  | | |
| Positive affect | -0.01 [-0.11, 0.08] | 1.51 | 0.00 | -0.02 [-0.14, 0.11] | 0.04 | 0.00 | -0.02 [-0.12, 0.08] | 0.90 | 0.00 |
|  |  |  |  |  |  |  |  |  |  |
| Feelings of empathy | | | | | | | | | |
|  |  |  |  |  |  |  |  |  |  |
| Empathy toward target | **0.09† [-0.01, 0.18]** | 4.46 | 32.76 | **0.14* [0.02, 0.27]** | 6.10* | 83.61 | 0.05 [-0.04, 0.15] | 3.08 | 2.54 |
|  |  |  |  |  |  |  |  |  |  |
| Positivity of impressions of target | | | | | | | | | |
|  |  |  |  |  |  |  |  |  |  |
| Impressions of target | 0.03 [-0.07, 0.12] | 13.10** | 77.10 | **0.16* [0.03, 0.28]** | 2.42 | 58.66 | **-0.12* [-0.21, -0.02]** | 5.59 | 46.34 |
| *Note.* Positive Cohen’s *d*s indicate greater reactivity toward Black targets (i.e., higher mean scores on a dependent measure when target was a Black individual), whereas negative Cohen’s *d*s indicate greater reactivity to White/European targets. Cohen’s *d* was computed using a fixed-effects model*.* CI = confidence interval; *Q* = Cochran’s *Q* and *I*^2^ =  *I*^2^ index used to test for and quantify the degree of heterogeneity in effect sizes, respectively. ^b^ = Cohen’s *d* based on a single study. † *p* < .10 * *p* < .05 ** *p* < .01 *** *p* < .001 | | | | | | | | | |

| Table S11 | | | | | | | |  | |  | | |
| --- | --- | --- | --- | --- | --- | --- | --- | --- | --- | --- | --- | --- |
| *Overall Effect of Target Type and Tests of Moderation for Affective Reactions, Empathy Toward Target, and Impressions of Target with Participants Suspicious about the Purpose of the Studies Excluded* | | | | | | | | | | | | |
|  |  | | |  | | Contrast | | | | | | |
| Measure | Effect across conditions | | | Omnibus moderation | | moderate vs. inclusion | | severe vs. inclusion | | severe vs. moderate | | |
|  | *d* 95% CI | *Q* | *I*^2^ | *QM*(*df*) | | *B* | *SE* | *B* | *SE* | *B* | *SE* | |
| Affective reactions | | | | | | | | | | | | |
| Guilt | **0.08* [0.02, 0.14]** | 16.93* | 46.85 | **13.29(2)**** | | **0.17*** | 0.08 | **0.25***** | 0.07 | 0.08 | 0.08 | |
|  |  |  |  |  |  |  |  |  |  |  |  | |
|  |  |  |  |  |  |  |  |  |  |  |  | |
| Anger | **0.06† [-0.00, 0.12]** | 34.25*** | 73.72 | **10.90(2)**** | | **0.16*** | 0.08 | **0.22**** | 0.07 | 0.06 | 0.08 | |
|  |  |  |  |  |  |  |  |  |  |  |  | |
|  |  |  |  |  |  |  |  |  |  |  |  | |
| Sadness | 0.05 [-0.01, 0.11] | 15.06† | 40.22 | **9.25(2)**** | | **0.15†** | 0.08 | **0.20**** | 0.07 | 0.06 | 0.08 | |
|  |  |  |  |  |  |  |  |  |  |  |  | |
|  |  |  |  |  |  |  |  |  |  |  |  | |
| Fear | **-**0.01 [-0.08, 0.06] | 7.08 | 43.50 | 4.32(2) | | -0.04 | 0.10 | **0.14†** | 0.08 | **0.17†** | 0.10 | |
|  |  |  |  |  |  |  |  |  |  |  |  | |
|  |  |  |  |  |  |  |  |  |  |  |  | |
| Positive affect | **-**0.02 [-0.08, 0.04] | 2.46 | 0.00 | 0.01(2) | | -0.004 | 0.08 | -0.01 | 0.07 | -0.002 | 0.08 | |
|  |  |  |  |  |  |  |  |  |  |  |  |  |
| Feelings of empathy | | | | | | | | | | | | |
|  |  |  |  |  |  |  |  |  |  |  |  | |
| Empathy toward target | **0.09** [0.03, 0.15]** | 14.93† | 39.71 | 1.29(2) | | 0.06 | 0.08 | -0.04 | 0.07 | -0.09 | 0.08 | |
|  |  |  |  |  |  |  |  |  |  |  |  | |
| Positivity of impressions of target | | | | | | | | | | | | |
|  |  |  |  |  |  |  |  |  |  |  |  | |
| Impressions of target | 0.002 [-0.06, 0.06] | 32.97*** | 72.70 | **11.86(2)**** | | 0.13 | 0.08 | **-0.14*** | 0.07 | **-0.27***** | 0.08 | |
| *Note.* Positive Cohen’s *d*s indicate greater reactivity toward Black targets (i.e., higher mean scores on a dependent measure when target was a Black individual), whereas negative Cohen’s *d*s indicate greater reactivity to White/European targets. Cohen’s *d* was computed using a fixed-effects model. CI = confidence interval; *Q* = Cochran’s *Q* and *I*^2^ =  *I*^2^ index used to test for and quantify the degree of heterogeneity in effect sizes, respectively; *QM* = omnibus test of moderator model coefficients; *df* = degrees of freedom; *SE* = standard error. † *p* < .10 * *p* < .05 ** *p* < .01 *** *p* < .001 | | | | | | | | | | | | |

| Table S12 | | | | | | | |
| --- | --- | --- | --- | --- | --- | --- | --- |
| *Overall Effect of Observed Treatment for Affective Reactions, Empathy Toward Target, and Impressions of Target for each Target Type with Participants Suspicious about the Purpose of the Studies Excluded* | | | | | | | |
| Measure/condition | White/European target | | |  | Black target | | |
|  | *d* 95% CI | *Q* | *I*^2^ |  | *d* 95% CI | *Q* | *I*^2^ |
| Affective reactions | | | | | | | |
| Guilt | 0.07 [-0.01, 0.16] | 20.01*** | 85.01 |  | **0.28*** [0.19, 0.36]** | 26.05*** | 88.48 |
|  |  |  |  |  |  |  |  |
|  |  |  |  |  |  |  |  |
| Anger | **0.30*** [0.22, 0.39]** | 16.45*** | 81.77 |  | **0.50*** [0.41, 0.59]** | 28.86*** | 89.61 |
|  |  |  |  |  |  |  |  |
|  |  | | |  |  | | |
| Sadness | **0.25*** [0.16, 0.34]** | 8.13* | 63.09 |  | **0.42*** [0.33, 0.51]** | 19.32*** | 84.47 |
|  |  |  |  |  |  |  |  |
|  |  | | |  |  | | |
| Fear | -0.04 [-0.14, 0.06] | 1.76 | 43.04 |  | 0.03 [-0.07, 0.14] | 2.38 | 57.91 |
|  |  |  |  |  |  |  |  |
|  |  | | |  |  | | |
| Positive affect | **-0.17*** [-0.26, -0.08]** | 26.31*** | 88.60 |  | **-0.18*** [-0.27, -0.10]** | 16.96*** | 82.31 |
|  |  |  |  |  |  |  |  |
| Feelings of empathy | | | | | | | |
|  |  |  |  |  |  |  |  |
| Empathy toward target | **0.50*** [0.42, 0.59]** | 21.44*** | 86.01 |  | **0.49*** [0.40, 0.58]** | 7.65† | 60.80 |
|  |  |  |  |  |  |  |  |
| Positivity of impressions of target | | | | | | | |
|  |  |  |  |  |  |  |  |
| Impressions of target | **0.12** [0.03, 0.21]** | 11.46** | 73.82 |  | 0.02 [-0.07, 0.11] | 9.01* | 66.72 |
| *Note.* Positive Cohen’s *d*s indicate greater reactivity to ostracism (i.e., higher mean scores on a dependent measure when the target was ostracized), whereas negative Cohen’s *d*s indicate greater reactivity to inclusion. Cohen’s *d* was computed using a fixed-effects model. The overall effect of observed treatment collapses across the moderate and severe ostracism conditions. CI = confidence interval; *Q* = Cochran’s *Q* and *I*^2^ =  *I*^2^ index used to test for and quantify the degree of heterogeneity in effect sizes, respectively. † *p* < .10 * *p* < .05 ** *p* < .01 *** *p* < .001 | | | | | | | |

| Table S13 | | | | | | | |
| --- | --- | --- | --- | --- | --- | --- | --- |
| *Overall Effect of Observed Treatment and Tests of Moderation for Affective Reactions, Empathy Toward Target, and Impressions of Target with Participants Suspicious about the Purpose of the Studies Excluded* | | | | | | | |
|  |  | | |  | | Contrast | |
| Measure | Effect across target type | | | Omnibus moderation | | Black vs. White/European target | |
|  | *d* 95% CI | *Q* | *I*^2^ | *QM*(*df*) | | *B* | *SE* |
| Affective reactions | | | | | | | |
| Guilt | **0.17*** [0.11, 0.24]** | 56.37*** | 87.58 | **10.31(1)**** | | **0.20**** | 0.06 |
|  |  |  |  |  |  |  |  |
|  |  |  |  |  |  |  |  |
| Anger | **0.40*** [0.34, 0.46]** | 54.77*** | 87.22 | **9.46(1)**** | | **0.20**** | 0.06 |
|  |  |  |  |  |  |  |  |
|  |  |  |  |  |  |  |  |
| Sadness | **0.33*** [0.27, 0.40]** | 35.10*** | 80.06 | **7.66(1)**** | | **0.18**** | 0.06 |
|  |  |  |  |  |  |  |  |
|  |  |  |  |  |  |  |  |
| Fear | -0.003 [-0.08, 0.07] | 5.04 | 40.45 | 0.91(1) | | 0.07 | 0.07 |
|  |  |  |  |  |  |  |  |
|  |  |  |  |  |  |  |  |
| Positive affect | **-0.18*** [-0.24, -0.11]** | 43.31*** | 83.84 | 0.05(1) | | -0.01 | 0.06 |
|  |  |  |  |  |  |  |  |
| Feelings of empathy | | | | | | | |
|  |  |  |  |  | |  |  |
| Empathy toward target | **0.50*** [0.44, 0.56]** | 29.12*** | 75.96 | 0.03(1) | | -0.01 | 0.06 |
|  |  |  |  |  |  |  |  |
| Positivity of impressions of target | | | | | | | |
|  |  |  |  |  | |  |  |
| Impressions of target | **0.07* [0.01, 0.13]** | 22.94** | 69.49 | 2.47(1) | | -0.10 | 0.06 |
| *Note.* Positive Cohen’s *d*s indicate greater reactivity to ostracism (i.e., higher mean scores on a dependent measure when the target was ostracized), whereas negative Cohen’s *d*s indicate greater reactivity to inclusion. Cohen’s *d* was computed using a fixed-effects model. The overall effect of observed treatment collapses across the moderate and severe ostracism conditions. CI = confidence interval; *Q* = Cochran’s *Q* and *I*^2^ =  *I*^2^ index used to test for and quantify the degree of heterogeneity in effect sizes, respectively; *QM* = omnibus test of moderator model coefficients; *df* = degrees of freedom; *SE* = standard error. * *p* < .05 ** *p* < .01 *** *p* < .001 | | | | | | | |

**9. Results of observed treatment × target type × participant sex ANOVAs for studies 3 to 4**

Tests of the effect of participant sex in Studies 3 and 4 focused on the three-way interaction because this interaction indicates if sex moderated the effect of most interest across our studies. A three-way interaction only emerged in Study 3 on impressions of the target, *F*(2, 1946) = 5.39, *p* = .005, $\text{η}_{\text{p}}^{\text{2}}$ = .006, 90% CI [.001, .012]. Women reported significantly more positive impressions of included Black targets relative to White targets (*p* < .001), whereas no such effect was evident for men. Both women’s and men’s impressions of outgroup and ingroup targets did not differ across the two ostracism conditions

**10. Assessment of social desirability**

In Studies 2 through 4 we measured participants’ social desirability concerns using the Balanced Inventory of Desirable Responding Short Form (BIDR-16 [8]). In Studies 2 and 3 participants completed both the self-deceptive enhancement (*α* = .80 and .77 in Study 2 and Study 3 respectively) and impression management (*α* = .78 and .74) subscales, whereas in Study 4 they only completed the impression management subscale (*α* = .74). These scales were completed at the end of each respective study. Our aim in incorporating these scales into the studies was to assess the degree to which participants’ responses on our main dependent measures may have been influenced by what they perceived to be the socially desirable response. Below we present estimates of the overall effect of target type and observed treatment as well as tests of moderation when controlling for participants’ impression management concerns in Studies 2 through 4. We included impression management rather than self-deceptive enhancement as a covariate because it was measured across more of the studies. The effect sizes (i.e., Cohen’s *d*) for these analyses were calculated from the estimated marginal means and their respective standard errors obtained from analyses of covariance (ANCOVA) conducted on each of our main dependent variables. Standard errors were converted to standard deviations for effect size computation. It should be noted that in Studies 2 and 4 we found that participants’ social desirability scores were affected by the manipulation, which complicates interpretation of the effect of our manipulation when controlling for the covariate.^2^ Consequently, the meta-analytic results computed when controlling for participants’ impression management concerns should be interpreted with caution.

| Table S14 | | | | | | | | | |
| --- | --- | --- | --- | --- | --- | --- | --- | --- | --- |
| *Overall Effect of Target Type for Affective Reactions, Empathy Toward Target, and Impressions of Target in Each Observed Treatment Condition Controlling for Participants’ Impression Management Concerns* | | | | | | | | | |
| Measure/condition | Inclusion  (Studies 1 to 4) | | | Moderate ostracism  (Studies 2 and 3 only) | | | Severe ostracism  (Studies 1 to 4) | | |
|  | *d* 95% CI | *Q* | *I*^2^ | *d* 95% CI | *Q* | *I*^2^ | *d* 95% CI | *Q* | *I*^2^ |
| Affective reactions | | | | | | | | | |
| Guilt | -0.07 [-0.16, 0.03] | 1.86 | 0.00 | 0.09 [-0.03, 0.22] | 0.34 | 0.00 | **0.20*** [0.10, 0.29]** | 1.93 | 0.00 |
|  |  |  |  |  |  |  |  |  |  |
|  |  |  |  |  |  |  |  |  |  |
| Anger | -0.06 [-0.16, 0.03] | 6.16† | 51.27 | 0.09 [-0.04, 0.22] | 8.21** | 87.82 | **0.19*** [0.10, 0.29]** | 11.92* | 74.84 |
|  |  |  |  |  |  |  |  |  |  |
|  |  | | |  | | |  | | |
| Sadness | -0.07 [-0.16, 0.03] | 2.42 | 0.00 | 0.07 [-0.05, 0.20] | 2.44 | 59.03 | **0.14** [0.05, 0.24]** | 0.70 | 0.00 |
|  |  |  |  |  |  |  |  |  |  |
|  |  | | |  | | |  | | |
| Fear | -0.05 [-0.16, 0.06] | 2.78† | 64.05 | -0.10^b^ [-0.25, 0.05] | -- | -- | 0.08 [-0.04, 0.19] | 0.13 | 0.00 |
|  |  |  |  |  |  |  |  |  |  |
|  |  | | |  | | |  | | |
| Positive affect | -0.01 [-0.11, 0.08] | 1.22 | 0.00 | -0.003 [-0.13, 0.12] | 0.24 | 0.00 | -0.02 [-0.11, 0.08] | 0.64 | 0.00 |
|  |  |  |  |  |  |  |  |  |  |
| Feelings of empathy | | | | | | | | | |
|  |  |  |  |  |  |  |  |  |  |
| Empathy toward target | 0.09† [-0.01, 0.18] | 3.73 | 19.54 | **0.15* [0.02, 0.28]** | 6.52* | 84.67 | 0.02 [-0.08, 0.11] | 4.03 | 25.65 |
|  |  |  |  |  |  |  |  |  |  |
| Positivity of impressions of target | | | | | | | | | |
|  |  |  |  |  |  |  |  |  |  |
| Impressions of target | 0.03 [-0.06, 0.12] | 11.03* | 72.80 | **0.17* [0.04, 0.29]** | 2.99† | 66.55 | **-0.13* [-0.23, -0.04]** | 4.76 | 37.04 |
| *Note.* Positive Cohen’s *d*s indicate greater reactivity toward Black targets (i.e., higher mean scores on a dependent measure when target was a Black individual), whereas negative Cohen’s *d*s indicate greater reactivity to White/European targets. Cohen’s *d* was computed using a fixed-effects model*.* CI = confidence interval; *Q* = Cochran’s *Q* and *I*^2^ =  *I*^2^ index used to test for and quantify the degree of heterogeneity in effect sizes, respectively. ^b^ = Cohen’s *d* based on a single study. † *p* < .10 * *p* < .05 ** *p* < .01 *** *p* < .001 | | | | | | | | | |

| Table S15 | | | | | | | |  | |  | | |
| --- | --- | --- | --- | --- | --- | --- | --- | --- | --- | --- | --- | --- |
| *Overall Effect of Target Type and Tests of Moderation for Affective Reactions, Empathy Toward Target, and Impressions of Target Controlling for Participants’ Impression Management Concerns* | | | | | | | | | | | | |
|  |  | | |  | | Contrast | | | | | | |
| Measure | Effect across conditions | | | Omnibus moderation | | moderate vs. inclusion | | severe vs. inclusion | | severe vs. moderate | | |
|  | *d* 95% CI | *Q* | *I*^2^ | *QM*(*df*) | | *B* | *SE* | *B* | *SE* | *B* | *SE* | |
| Affective reactions | | | | | | | | | | | | |
| Guilt | **0.07* [0.01, 0.13]** | 19.04* | 52.74 | **14.90(2)***** | | **0.16*** | 0.08 | **0.26***** | 0.07 | 0.10 | 0.08 | |
|  |  |  |  |  |  |  |  |  |  |  |  | |
|  |  |  |  |  |  |  |  |  |  |  |  | |
| Anger | **0.07* [0.01, 0.13]** | 40.20*** | 77.61 | **13.91(2)***** | | **0.15†** | 0.08 | **0.25**** | 0.07 | 0.10 | 0.08 | |
|  |  |  |  |  |  |  |  |  |  |  |  | |
|  |  |  |  |  |  |  |  |  |  |  |  | |
| Sadness | 0.05 [-0.01, 0.10] | 15.61† | 42.36 | **10.06(2)**** | | **0.14†** | 0.08 | **0.21**** | 0.07 | 0.07 | 0.08 | |
|  |  |  |  |  |  |  |  |  |  |  |  | |
|  |  |  |  |  |  |  |  |  |  |  |  | |
| Fear | **-**0.01 [-0.08, 0.06] | 7.18 | 44.30 | 4.27(2) | | -0.05 | 0.10 | 0.13 | 0.08 | **0.18†** | 0.10 | |
|  |  |  |  |  |  |  |  |  |  |  |  | |
|  |  |  |  |  |  |  |  |  |  |  |  | |
| Positive affect | **-**0.01 [-0.07, 0.05] | 2.15 | 0.00 | 0.04(2) | | 0.01 | 0.08 | -0.01 | 0.07 | -0.02 | 0.08 | |
|  |  |  |  |  |  |  |  |  |  |  |  |  |
| Feelings of empathy | | | | | | | | | | | | |
|  |  |  |  |  |  |  |  |  |  |  |  | |
| Empathy toward target | **0.07** [0.02, 0.13]** | 17.07* | 47.27 | 2.78(2) | | 0.06 | 0.08 | -0.07 | 0.07 | -0.13 | 0.08 | |
|  |  |  |  |  |  |  |  |  |  |  |  | |
| Positivity of impressions of target | | | | | | | | | | | | |
|  |  |  |  |  |  |  |  |  |  |  |  | |
| Impressions of target | -0.004 [-0.06, 0.06] | 33.35*** | 73.01 | **14.57(2)***** | | **0.14†** | 0.08 | **-0.16*** | 0.07 | **-0.30***** | 0.08 | |
| *Note.* Positive Cohen’s *d*s indicate greater reactivity toward Black targets (i.e., higher mean scores on a dependent measure when target was a Black individual), whereas negative Cohen’s *d*s indicate greater reactivity to White/European targets. Cohen’s *d* was computed using a fixed-effects model. CI = confidence interval; *Q* = Cochran’s *Q* and *I*^2^ =  *I*^2^ index used to test for and quantify the degree of heterogeneity in effect sizes, respectively; *QM* = omnibus test of moderator model coefficients; *df* = degrees of freedom; *SE* = standard error. † *p* < .10 * *p* < .05 ** *p* < .01 *** *p* < .001 | | | | | | | | | | | | |

| Table S16 | | | | | | | |
| --- | --- | --- | --- | --- | --- | --- | --- |
| *Overall Effect of Observed Treatment for Affective Reactions, Empathy Toward Target, and Impressions of Target for each Target Type Controlling for Participants’ Impression Management Concerns* | | | | | | | |
| Measure/condition | White/European target | | |  | Black target | | |
|  | *d* 95% CI | *Q* | *I*^2^ |  | *d* 95% CI | *Q* | *I*^2^ |
| Affective reactions | | | | | | | |
| Guilt | 0.06 [-0.02, 0.15] | 21.78*** | 86.23 |  | **0.28*** [0.19, 0.37]** | 26.58*** | 88.71 |
|  |  |  |  |  |  |  |  |
|  |  |  |  |  |  |  |  |
| Anger | **0.29*** [0.21, 0.38]** | 16.97*** | 82.32 |  | **0.53*** [0.44, 0.62]** | 34.96*** | 91.42 |
|  |  |  |  |  |  |  |  |
|  |  | | |  |  | | |
| Sadness | **0.24*** [0.15, 0.33]** | 9.55* | 68.57 |  | **0.42*** [0.34, 0.51]** | 19.57*** | 84.67 |
|  |  |  |  |  |  |  |  |
|  |  | | |  |  | | |
| Fear | -0.04 [-0.15, 0.06] | 2.75† | 63.67 |  | 0.03 [-0.07, 0.13] | 3.30† | 69.66 |
|  |  |  |  |  |  |  |  |
|  |  | | |  |  | | |
| Positive affect | **-0.18*** [-0.26, -0.09]** | 26.50*** | 88.68 |  | **-0.17*** [-0.26, -0.09]** | 17.71*** | 83.06 |
|  |  |  |  |  |  |  |  |
| Feelings of empathy | | | | | | | |
|  |  |  |  |  |  |  |  |
| Empathy toward target | **0.51*** [0.42, 0.60]** | 18.94*** | 84.16 |  | **0.47*** [0.38, 0.56]** | 9.65* | 68.90 |
|  |  |  |  |  |  |  |  |
| Positivity of impressions of target | | | | | | | |
|  |  |  |  |  |  |  |  |
| Impressions of target | **0.12** [0.03, 0.21]** | 13.67** | 78.06 |  | 0.01 [-0.07, 0.10] | 8.31* | 63.91 |
| *Note.* Positive Cohen’s *d*s indicate greater reactivity to ostracism (i.e., higher mean scores on a dependent measure when the target was ostracized), whereas negative Cohen’s *d*s indicate greater reactivity to inclusion. Cohen’s *d* was computed using a fixed-effects model. The overall effect of observed treatment collapses across the moderate and severe ostracism conditions. CI = confidence interval; *Q* = Cochran’s *Q* and *I*^2^ =  *I*^2^ index used to test for and quantify the degree of heterogeneity in effect sizes, respectively. † *p* < .10 * *p* < .05 ** *p* < .01 *** *p* < .001 | | | | | | | |

| Table S17 | | | | | | | |
| --- | --- | --- | --- | --- | --- | --- | --- |
| *Overall Effect of Observed Treatment and Tests of Moderation for Affective Reactions, Empathy Toward Target, and Impressions of Target Controlling for Participants’ Impression Management Concerns* | | | | | | | |
|  |  | | |  | | Contrast | |
| Measure | Effect across target type | | | Omnibus moderation | | Black vs. White/European target | |
|  | *d* 95% CI | *Q* | *I*^2^ | *QM*(*df*) | | *B* | *SE* |
| Affective reactions | | | | | | | |
| Guilt | **0.17*** [0.11, 0.23]** | 60.26*** | 88.38 | **11.91(1)***** | | **0.22***** | 0.06 |
|  |  |  |  |  |  |  |  |
|  |  |  |  |  |  |  |  |
| Anger | **0.41*** [0.35, 0.47]** | 65.57*** | 89.32 | **13.63(1)***** | | **0.23**** | 0.06 |
|  |  |  |  |  |  |  |  |
|  |  |  |  |  |  |  |  |
| Sadness | **0.33*** [0.27, 0.39]** | 37.30*** | 81.24 | **8.19(1)**** | | **0.18**** | 0.06 |
|  |  |  |  |  |  |  |  |
|  |  |  |  |  |  |  |  |
| Fear | -0.01 [-0.08, 0.06] | 7.03† | 57.32 | 0.98(1) | | 0.07 | 0.07 |
|  |  |  |  |  |  |  |  |
|  |  |  |  |  |  |  |  |
| Positive affect | **-0.17*** [-0.24, -0.11]** | 44.21*** | 84.17 | 0.0001(1) | | 0.0005 | 0.06 |
|  |  |  |  |  |  |  |  |
| Feelings of empathy | | | | | | | |
|  |  |  |  |  | |  |  |
| Empathy toward target | **0.49*** [0.43, 0.55]** | 28.87*** | 75.75 | 0.28(1) | | -0.03 | 0.06 |
|  |  |  |  |  |  |  |  |
| Positivity of impressions of target | | | | | | | |
|  |  |  |  |  | |  |  |
| Impressions of target | **0.07* [0.01, 0.13]** | 24.83*** | 71.81 | **2.84(1)†** | | **-0.11†** | 0.06 |
| *Note.* Positive Cohen’s *d*s indicate greater reactivity to ostracism (i.e., higher mean scores on a dependent measure when the target was ostracized), whereas negative Cohen’s *d*s indicate greater reactivity to inclusion. Cohen’s *d* was computed using a fixed-effects model. The overall effect of observed treatment collapses across the moderate and severe ostracism conditions. CI = confidence interval; *Q* = Cochran’s *Q* and *I*^2^ =  *I*^2^ index used to test for and quantify the degree of heterogeneity in effect sizes, respectively; *QM* = omnibus test of moderator model coefficients; *df* = degrees of freedom; *SE* = standard error. † *p* < .10 * *p* < .05 ** *p* < .01 *** *p* < .001 | | | | | | | |

**Footnotes**

1. 90% confidence intervals around partial $\text{η}^{\text{2}}$ squared are equivalent to 95% confidence intervals around *d* [1].

2. In Study 2 a marginally significant interaction emerged on the impression management subscale, *F*(1, 918) = 2.82, *p* = .060, $\text{η}_{\text{p}}^{\text{2}}$ = .003, 90% CI [.000, .012]. Simple effects tests revealed a significant effect of target type in the moderate ostracism condition, such that participants reported greater impression management concerns after observing a White/European target (*M* = 4.54, *SD* = 1.05) compared to a Black target (*M* = 4.25, *SD* = 0.99) be moderately ostracized, *F*(1, 918) = 4.86, *p* = .028, $\text{η}_{\text{p}}^{\text{2}}$ = .005, 90% CI [.000, .016]. In Study 4 there was a significant interaction on impression management, *F*(1, 1226) = 5.95, *p* = .015, $\text{η}_{\text{p}}^{\text{2}}$ = .005, 90% CI [.000, .013]. The simple effect of target type was significant in the inclusion condition, with impression management concerns being greater after participants observed a White/European target (*M* = 4.47, *SD* = 1.10) compared to a Black target (*M* = 4.25, *SD* = 1.01) be included, *F*(1, 1226) = 5.96, *p* = .015, $\text{η}_{\text{p}}^{\text{2}}$ = 005, 90% CI [.000, .013]. In Studies 2 and 4 no effects emerged on the self-deceptive enhancement subscale and no effects were evident on any subscale in Study 3.

**References**

1. Steiger JH. Beyond the F test: Effect size confidence intervals and tests of close fit in the analysis of variance and contrast analysis. Psychol Methods. 2004;9(2): 164-182. pmid: 15137887

2. Wrightsman LS. Assumptions about human nature: A social-psychological

approach. Monterey, CA: Brooks/Cole Publishing; 1974.

3. Wrightsman LS. Interpersonal trust and attitudes toward human nature. In J. P.

Robinson JP, Shaver PR, Wrightsman LS, editors. Measures of social

psychological attitudes, Vol. 1. Measures of personality and social psychological

attitudes. San Diego, CA: Academic Press; 1991, pp. 373-412.

4. Survey Research Center. 1964 Election Study. Ann Arbor, MI: Inter-University Consortium for Political Research, University of Michigan; 1969.

5. Gillath O, Hart J, Noftle EE, Stockdale GD. Development and validation of a state adult attachment measure (SAAM). J Res Pers. 2009;43(3): 361-373.

6. Branscombe NR, Slugoski B, Kappen DM. The measurement of collective guilt: What it is and what it is not. In Branscombe NR, Doosje B, editors. Collective guilt: International perspectives. Cambridge, UK: Cambridge University Press; 2004. pp. 16-34.

7. Piff PK, Martinez AG, Keltner D. Me against we: In-group transgression, collective shame, and in-group-directed hostility. Cogn Emot. 2012;26(4): 634–49. pmid: 21827331

8. Hart CM, Ritchie TD, Hepper EG, Gebauer JE. The balanced inventory of desirable responding short form (BIDR-16). Sage Open. 2015;5(4): 1-9.
